# A Multimodal Fake News Detection Model Based on Adaptive Binary Osprey Optimization Algorithm and Cross-Modal Disentangled Fusion

**DOI:** 10.3390/biomimetics11090601

**Published:** 2026-08-23

**Authors:** Xu Dai, Guoqiang Lu, Jiaxue Li

**Affiliations:** 1 School of Finance, Jilin University of Finance and Economics, Changchun 130117, China; daixusissi@jlufe.edu.cn; 2 Jilin Provincial Key Laboratory of Fintech, Changchun 130117, China; 3 School of Health Management, Mudanjiang Medical University, Mudanjiang 157011, China; luguoqiang_123@163.com; 4 Institute of National Development and Security Studies, Jilin University, Changchun 130012, China

**Keywords:** multimodal fake news detection, cross-modal relation disentanglement, adaptive semantic fusion, swarm intelligence optimization

## Abstract

With the rapid growth of social media, online news has become increasingly multimodal, combining textual and visual information, posing new challenges for fake news detection. Existing methods often suffer from redundant features, distribution differences across modalities, and insufficient modeling of semantic interactions. To address these issues, this paper proposes an Adaptive Binary Osprey Optimization Algorithm and Cross-modal Disentangled Fusion model (ABOOA-CDF). First, an Adaptive Binary Osprey Optimization Algorithm (ABOOA) is developed for multimodal feature selection by integrating chaotic initialization, adaptive search, and binary mapping strategies to identify informative feature subsets. Then, a Cross-modal Relation Disentanglement Module (CRDM) is introduced to decompose multimodal representations into shared, discrepant, and complementary components, thereby enhancing semantic relationship modeling. Furthermore, an Adaptive Semantic Fusion Module (ASFM) dynamically learns fusion weights to generate discriminative multimodal representations. Experimental results demonstrate that ABOOA-CDF effectively improves detection performance. Compared with MFO and OOA, the proposed method achieves Accuracy improvements of 1.02 and 2.66 percentage points, respectively, verifying its effectiveness in feature optimization, cross-modal relation modeling, and semantic fusion.

## 1. Introduction

With the rapid development of Internet technologies and social media platforms, the creation and dissemination of news have significantly changed. News can now spread rapidly across online websites, enabling information to reach a large audience quickly. However, the openness and convenience of online communication have also accelerated the dissemination of fake news, posing serious threats to public opinion, social stability, and information security. Consequently, accurate fake news detection has become an important research topic in natural language processing, multimedia analysis, and intelligent information processing.

Early fake news detection methods mainly rely on textual information and use handcrafted features alongside traditional machine learning classifiers. Although these approaches achieve reasonable performance in specific scenarios, they are limited in modeling complex semantics, contextual dependencies, and implicit logical relationships. With the advancement of deep learning, neural representation learning has significantly improved the extraction of semantic features from text, providing more effective solutions for fake news detection [1]. Nevertheless, real-world news often comprises multiple modalities, such as text and images. Depending solely on textual information is therefore insufficient to capture the complete characteristics of news content [2].

The increasing prevalence of multimodal news has promoted extensive research on multimodal fake news detection. Existing approaches typically integrate textual and visual representations and exploit cross-modal interactions to determine news credibility, thereby alleviating the limitations of single-modal methods [3,4]. Despite these developments, text and image modalities differ significantly in their representations, semantic patterns, and feature distributions. The interaction between them may simultaneously contain shared semantic content, complementary clues, and inconsistent information. Although existing approaches generally capture such relationships through feature fusion or cross-modal interaction mechanisms, they seldom explicitly characterize various semantic relationship types. Consequently, the discriminative information contained in multimodal data may not be fully utilized.

Recent progress in cross-modal representation learning and contrastive learning has further strengthened semantic alignment between different modalities [5]. In addition, multimodal contrastive learning enhanced with external knowledge has improved the quality of representations in complex news scenarios [6]. Beyond semantic alignment, disentanglement-based approaches have been explored to separate shared and modality-specific information in multimodal data [7]. Nevertheless, existing multimodal fake news detection methods still focus on overall semantic consistency, neglecting fine-grained cross-modal relationships. This limitation reduces their ability to capture discriminative cues embedded in multimodal content. Therefore, effectively disentangling different types of cross-modal relationships remains a key challenge in multimodal fake news detection.

Another challenge arises from the high dimensionality of multimodal representations. After feature extraction, textual and visual features often contain considerable redundancy and weakly relevant information. These redundant features increase computational complexity and may degrade classification performance. Swarm intelligence optimization algorithms have been widely adopted for feature selection due to their strong global search capabilities and gradient-free optimization. Representative methods, such as the Whale Optimization Algorithm (WOA) and Sparrow Search Algorithm (SSA), have demonstrated promising performance in feature subset optimization [8,9]. More recently, newly developed nature-inspired optimization algorithms have further expanded the solution space for complex optimization problems [10]. However, existing algorithms still suffer from unstable search behavior and premature convergence when solving high-dimensional binary feature selection problems, limiting their effectiveness in multimodal fake news detection.

To address these issues, this paper proposes an Adaptive Binary Osprey Optimization Algorithm and Cross-modal Disentangled Fusion (ABOOA-CDF) for multimodal fake news detection. The proposed framework combines adaptive feature optimization with fine-grained cross-modal relation modeling to improve the quality of multimodal representations and enhance detection performance.

The main contributions of this paper are summarized as follows.

(1) An Adaptive Binary Osprey Optimization Algorithm (ABOOA) is proposed for high-dimensional binary feature selection. By incorporating circle chaotic initialization, an adaptive search strategy, and a binary mapping mechanism, the proposed algorithm improves search efficiency and selects more informative feature subsets.
(2) A Cross-modal Relation Disentanglement Module (CRDM) is developed to model different semantic relationships between text and images explicitly. The module decomposes cross-modal representations into shared, discrepant, and complementary components, reducing interference among different types of semantic information.
(3) An Adaptive Semantic Fusion Module (ASFM) is designed to dynamically assign fusion weights according to the semantic characteristics of each sample, enabling more effective integration of multimodal representations.
(4) Extensive experiments are conducted on real-world multimodal fake news datasets. The proposed method achieves the best overall performance, demonstrating the effectiveness of the proposed framework.

## 2. Related Work

### 2.1. Unimodal Fake News Detection

Early fake news detection mainly relies on textual content and exploits linguistic patterns, propagation characteristics, and handcrafted statistical features to distinguish fake news from real news. Conventional approaches typically extract features from word frequency, topic distribution, sentiment polarity, and user behavior, and then employ machine learning classifiers such as Support Vector Machines (SVM), Decision Trees, and Random Forests for classification. Although these methods are computationally efficient and easy to implement, their performance heavily depends on manually designed features. Consequently, they often fail to capture deep semantic representations and complex contextual dependencies, especially when processing fake news with diverse writing styles and implicit semantic patterns [11,12].

The rapid development of deep learning has significantly advanced unimodal fake news detection. Neural representation learning enables automatic extraction of latent patterns from news content and propagation behavior, reducing reliance on manual feature engineering. Deep neural networks can model complex nonlinear relationships and therefore provide more discriminative representations for fake news identification [13]. In parallel, approaches that incorporate user interactions and propagation structures further improve detection performance by exploiting the dynamic dissemination process of online news [14].

More recently, pretrained language models have substantially enhanced text-based fake news detection. Contextual representations learned from large-scale corpora enable dynamic interpretation of word meanings across contexts, thereby improving the modeling of complex semantics and long-range dependencies. Transformer-based pretrained models have demonstrated strong generalization ability in text classification and have gradually become the foundation of modern fake news detection systems [15]. Building upon this framework, RoBERTa further refines the pretraining strategy and optimization process, producing more robust language representations for complex classification tasks [16].

Despite these advances, text-only approaches still exhibit several limitations. News articles can be intentionally manipulated through paraphrasing, implicit expressions, or semantic reconstruction, making authenticity difficult to determine solely from textual evidence. Furthermore, online news generally combines text with images and other complementary media. Ignoring these heterogeneous sources prevents models from fully exploiting the available evidence. As a result, unimodal methods often struggle in real-world scenarios and require additional multimodal information to improve detection accuracy [17].

Overall, unimodal fake news detection has evolved from handcrafted feature engineering to deep representation learning and, more recently, pretrained language modeling. These developments have established a solid foundation for subsequent research. Although online news increasingly incorporates multiple modalities, relying solely on textual information is no longer sufficient. Therefore, integrating heterogeneous data sources has become an important direction for improving fake news detection.

### 2.2. Multimodal Fake News Detection

The rapid growth of social media has transformed online news from pure text into multimodal content that combines text, images, videos, and other media. Text describes events, facts, and opinions, whereas images provide complementary visual evidence, such as people, scenes, and contextual information. By jointly exploiting these heterogeneous sources, multimodal fake news detection provides a more comprehensive understanding of news content than text-only approaches. Therefore, it has become a major research direction in fake news detection.

Early fake news detection methods mainly rely on textual representations or social propagation information, where deep learning models are developed to capture semantic features and structural patterns from single-source data [18,19,20]. However, these approaches struggle to leverage complementary information across modalities, motivating the development of multimodal fake news detection methods.

With the development of multimodal fake news detection, researchers focus on learning joint representations of textual and visual content. These approaches extract features from different modalities and project them into a shared feature space for credibility assessment. The Event Adversarial Neural Network (EANN) introduces event adversarial learning to reduce the influence of event distribution bias, thereby improving generalization across unseen events [21]. Subsequently, attention shifts to visual information. Multi-domain visual modeling methods explore object regions, scene context, and visual semantics to enhance visual representations and improve detection performance in complex news scenarios [22].

As cross-modal learning advances, research has gradually shifted from joint representation learning to semantic relationship modeling. Text and images may exhibit semantic consistency, conflicts, or missing correspondence. Modeling all interactions within a unified representation is often insufficient to capture these diverse relationships. SAFE improves the identification of inconsistent image–text pairs through a similarity-aware mechanism [23]. Multi-view representation learning further models cross-modal interactions from textual semantics, visual patterns, and high-level semantic perspectives, leading to more discriminative multimodal representations [24]. In addition, similarity-aware prompt learning strengthens semantic alignment between modalities by optimizing prompt representations, further improving cross-modal understanding [25].

Recent advances in vision-language pretraining have driven multimodal fake news detection toward knowledge-enhanced frameworks. Prompt-based methods effectively leverage prior knowledge embedded in vision-language models, achieving better performance in few-shot settings and early-stage fake news detection [26]. Emotion-aware prompt learning further integrates textual sentiment, visual semantics, and cross-modal interactions to improve the modeling of complex semantic relationships [27]. Meanwhile, adaptive feature selection, multimodal large language models, and cross-modal entity consistency have been incorporated into fake news detection. These techniques improve misinformation recognition by dynamically selecting informative features, incorporating external knowledge, and modeling entity correspondence across modalities [28,29,30]. Recent studies have further extended multimodal fake news detection to knowledge-enriched and multi-domain settings [31]. More recently, fine-grained attribution analysis and multi-perspective evidence verification have further enhanced reasoning capability and prediction reliability [32,33].

Overall, multimodal fake news detection has evolved from shallow feature fusion to fine-grained cross-modal relationship modeling and knowledge-enhanced learning, leading to substantial improvements in detection performance. Nevertheless, several challenges persist. Most existing methods still rely on a unified representation space, making it difficult to distinguish shared, discrepant, and complementary semantic information. In addition, multimodal representations often contain redundant features that reduce learning efficiency. The relative importance of different modalities also varies across news samples, whereas existing fusion strategies generally employ fixed or insufficiently adaptive mechanisms. Therefore, effective feature selection, fine-grained modeling of heterogeneous cross-modal relationships, and adaptive fusion of multimodal evidence remain important directions for future research.

## 3. Methodology

**Figure 1:**
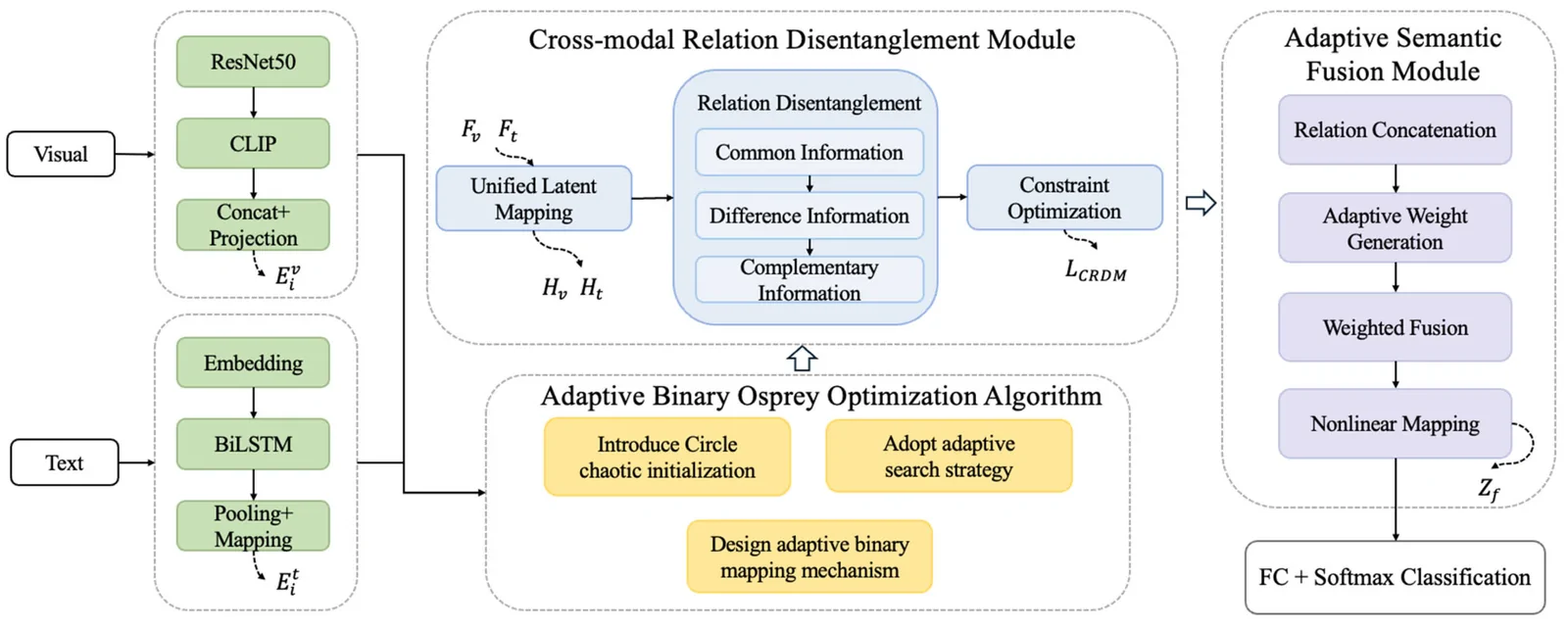
Framework of the Proposed ABOOA-CDF Model.

This paper proposes a multimodal fake news detection model based on the Adaptive Binary Osprey Optimization Algorithm and Cross-modal Disentangled Fusion (ABOOA-CDF). The framework consists of three sequential stages: feature optimization, relation disentanglement, and semantic fusion. First, the Adaptive Binary Osprey Optimization Algorithm (ABOOA) is employed to select informative features from both image and text modalities. Circle chaotic initialization, an adaptive search strategy, and a binary mapping mechanism are incorporated to optimize the feature subset while reducing redundant information. Next, the Cross-modal Relation Disentanglement Module (CRDM) projects visual and textual features into a unified representation space. The cross-modal information is then decomposed into three complementary components: shared, discrepant, and complementary. This disentangled representation improves the discrimination of semantic relationships across different modalities. Finally, the Adaptive Semantic Fusion Module (ASFM) dynamically assigns fusion weights to the three relation components according to their contributions. The resulting fused representation is fed into a fully connected layer followed by a Softmax classifier to predict the authenticity of the news. The overall architecture of the proposed model is illustrated in Figure 1.

### 3.1. Multimodal Feature Extraction

Multimodal fake news detection requires both textual semantics and visual evidence. The text modality conveys news, opinions, and emotional expressions, whereas the image modality provides complementary information, such as scenes, objects, and visual context. To provide reliable inputs for subsequent feature selection and cross-modal relation modeling, text and image features are extracted separately.

#### 3.1.1. Text Feature Extraction

News articles exhibit strong contextual dependencies, where the meaning of each word is influenced by its surrounding context. To capture bidirectional semantic information, a Bidirectional Long Short-Term Memory (BiLSTM) network is adopted for text encoding [34].

Given the tokenized text of the i-th news sample, the sequence is defined as

(1) $$\begin{array}{c} \begin{array}{cccc} & P_{i}=\left[w_{i,1},w_{i,2},\ldots ,w_{i,L}\right] & & \end{array} \end{array}$$

where $P_{i}$ denotes the text sequence, $w_{i,k}$ represents the k-th token, and L is the sequence length. Each token is first mapped into a continuous embedding vector.

(2) $$\begin{array}{c} e_{i,k}=Emb\left(w_{i,k}\right) \end{array}$$

where $e_{i,k}$ is the embedding of $w_{i,k}$, and Emb(⋅) denotes the embedding function.

The forward and backward LSTM networks then process the embedding sequence.

(3) $${\vec{h}}_{i,k}={\mathrm{LSTM}}_{f}(e_{i,k},{\vec{h}}_{i,k-1})$$

(4) $${\overset{\leftarrow }{h}}_{i,k}={\mathrm{LSTM}}_{b}(e_{i,k},{\overset{\leftarrow }{h}}_{i,k+1})$$

where ${\vec{h}}_{i,k}$ and ${\overset{\leftarrow }{h}}_{i,k}$ denote the forward and backward hidden states, respectively. The hidden representations from both directions are concatenated to obtain the contextual representation.

(5) $$h_{i,k}=[{\vec{h}}_{i,k};{\overset{\leftarrow }{h}}_{i,k}]$$

where [;] denotes feature concatenation. To generate a global representation of the entire text, average pooling is applied over all hidden states.

(6) $$g_{i}^{t}=\frac{1}{L}\sum_{k=1}^{L}h_{i,k}$$

$g_{i}^{t}$ denotes the global textual representation of the i-th news sample. Finally, a nonlinear transformation is employed to obtain the text feature.

(7) $$E_{i}^{t}=\phi (W_{t}^{0}\cdot g_{i}^{t}+b_{t}^{0})$$

where $E_{i}^{t}$ is the extracted text feature, $W_{t}^{0}$ and $b_{t}^{0}$ are learnable parameters, and ϕ(⋅) denotes the activation function.

#### 3.1.2. Image Feature Extraction

Images provide visual evidence that complements textual information. To preserve both low-level visual structures and high-level semantic information, ResNet50 and CLIP are jointly employed for image encoding. ResNet50 extracts structural visual patterns such as scenes, textures, and objects [35], while CLIP produces image representations aligned with the shared vision-language semantic space [36].

Let $I_{i}$ denote the image corresponding to the i-th news sample. Deep visual features are first extracted using ResNet50 without its final classification layer.

(8) $$r_{i}=\mathrm{GAP}(\mathrm{ResNet}50(I_{i}))$$

where $r_{i}$ denotes the visual feature extracted by ResNet50, and GAP(⋅) represents global average pooling. The CLIP image encoder then encodes the image.

(9) $$c_{i}={\mathrm{CLIP}}_{img}(I_{i})$$

$c_{i}$ denotes the semantic image representation generated by CLIP. Since these two feature types have different distributions and dimensionalities, nonlinear transformations are first applied.

(10) $${\hat{r}}_{i}=\phi (W_{r}\cdot r_{i}+b_{r})$$

(11) $${\hat{c}}_{i}=\phi (W_{c}\cdot c_{i}+b_{c})$$

${\hat{r}}_{i}$ and ${\hat{c}}_{i}$ denote the transformed ResNet50 and CLIP features, respectively. $W_{r}$, $W_{c}$, $b_{r}$, and $b_{c}$ are learnable parameters. The transformed features are concatenated to form the joint image representation.

(12) $$g_{i}^{v}=[{\hat{r}}_{i};{\hat{c}}_{i}]$$

$g_{i}^{v}$ denotes the fused visual representation. A projection layer is then applied to obtain the final image feature.

(13) $$E_{i}^{v}=\phi (W_{v}^{0}\cdot g_{i}^{v}+b_{v}^{0})$$

where $E_{i}^{v}$ is the extracted image feature, and $W_{v}^{0}$ and $b_{v}^{0}$ are learnable parameters.

In summary, BiLSTM is employed to extract the textual feature $E_{i}^{t}$, while ResNet50 and CLIP are jointly used to obtain the image feature $E_{i}^{v}$. These features are fed into the ABOOA feature selection module to remove redundant dimensions and retain more discriminative multimodal information. The selected text and image features, denoted as $F_{t}$ and $F_{v}$, are subsequently used as inputs to the Cross-modal Relation Disentanglement Module (CRDM).

### 3.2. Osprey Optimization Algorithm (OOA)

The Osprey Optimization Algorithm (OOA) is a swarm intelligence optimization method proposed by Dehghani et al. based on the hunting behavior of ospreys in nature [37]. OOA simulates the processes of prey detection, target diving, and prey capture to achieve global exploration and local exploitation in the search space. The algorithm consists of two stages: global exploration (prey localization) and local exploitation (prey capture).

#### 3.2.1. Global Exploration Stage (Prey Localization)

At the beginning of hunting, an osprey circles above the water to search for prey. After identifying a promising region, it moves toward the target. In OOA, each osprey represents a search agent, while the prey location corresponds to a potential optimal solution. During optimization, each agent updates its position according to a selected prey candidate and its current position.

Step 1: Construct the Candidate Prey Set

For the i-th osprey, all individuals with better fitness than the current solution are collected to form the candidate prey set.

(14) $$S_{i}=\{X_{k}|F(X_{k})<F(X_{i}),k\in \{1,2,\ldots ,N\},k\neq i\}$$

where N denotes the population size and F(⋅) is the objective function. If $S_{i}$ is empty, one individual other than $X_{i}$ is randomly selected from the population as the prey.

Step 2: Select a Prey Position

A prey position, denoted as $FP_{i}$, is randomly selected from the candidate prey set $S_{i}$.

Step 3: Update the Position

(15) $$x_{i,j}^{\mathrm{new},P1}=x_{i,j}+r_{i,j}\cdot \left(FP_{i,j}-I_{i,j}\cdot x_{i,j}\right),i=1,2,\ldots ,N;j=1,2,\ldots ,m$$

where $x_{i,j}$ is the current position of the i-th osprey in the j-th dimension, $FP_{i,j}$ is the corresponding coordinate of the selected prey, $r_{i,j}$ in [0, 1] is a random number, $I_{i,j}$ in {1, 2} is a random integer controlling the movement direction, and m denotes the problem dimension. This update guides the search agent toward a promising region.

Step 4: Greedy Selection

The updated solution is accepted only if it improves the objective value.

(16) $$X_{i}\leftarrow \left\{\begin{array}{cc} X_{i}^{\mathrm{new},P1}, & \mathrm{if}\;F(X_{i}^{\mathrm{new},P1})<F(X_{i})\\ X_{i}, & \mathrm{otherwise} \end{array}\right.$$

This greedy strategy prevents deterioration of the population fitness.

#### 3.2.2. Local Exploitation Stage (Prey Capture)

After locating the prey, the osprey performs fine adjustments before completing the capture. In the optimization process, this behavior is modeled as a local search around the current solution. As the iteration proceeds, the search step gradually decreases, allowing the algorithm to shift from broad exploration to refined exploitation. The position update in the j-th dimension is defined as

(17) $$x_{i,j}^{\mathrm{new},P2}=x_{i,j}+\frac{r_{i,j}\cdot (ub_{j}-lb_{j})+lb_{j}}{t},i=1,2,\ldots ,N;j=1,2,\ldots ,m$$

where $x_{i,j}^{\mathrm{new},P2}$ is the updated position, $lb_{j}$ and $ub_{j}$ denote the lower and upper bounds of the j-th dimension, $r_{i,j}\;\mathrm{in}\;\left[0,1\right]$ is a random number, and t is the current iteration number. The factor $\frac{1}{t}$ gradually reduces the perturbation amplitude, thereby strengthening local exploitation in the later search stage. After the update, greedy selection is again applied.

(18) $$X_{i}\leftarrow \left\{\begin{array}{cc} X_{i}^{\mathrm{new},P2}, & \mathrm{if}\;F(X_{i}^{\mathrm{new},P2})<F(X_{i})\\ X_{i}, & \mathrm{otherwise} \end{array}\right.$$

where $X_{i}$ and $X_{i}^{\mathrm{new},P2}$ denote the solution vectors before and after the update, respectively, and F(⋅) is the objective function. This strategy ensures that the population’s best fitness is preserved during optimization.

Overall, OOA combines global exploration with local exploitation to balance search diversity and convergence. The exploration stage rapidly identifies promising regions, whereas the exploitation stage continuously refines candidate solutions. Despite this balance, the original OOA still has several limitations. Its initialization relies on random sampling, and its search mechanism is mainly designed for continuous optimization. Consequently, its performance may deteriorate in high-dimensional binary feature selection tasks. These limitations motivate the improvements introduced in the proposed optimization algorithm for multimodal feature selection.

### 3.3. Adaptive Binary Osprey Optimization Algorithm (ABOOA)

Although OOA demonstrates strong optimization capability in continuous optimization problems, its direct application to high-dimensional feature selection remains challenging. To improve population quality, search efficiency, and binary feature selection performance, this study proposes an Adaptive Binary Osprey Optimization Algorithm (ABOOA) based on the original OOA. The proposed algorithm incorporates a circle chaotic initialization strategy, an adaptive search strategy, and an adaptive binary mapping mechanism. These improvements enhance global exploration, local exploitation, and binary optimization in discrete feature spaces.

Unlike the original OOA, ABOOA maintains both a continuous search position and a binary feature selection vector for each search agent. The continuous position is updated during the optimization process, whereas the binary vector represents the selected feature subset for fitness evaluation. Let the population size be N, and the original feature dimension be m. The continuous search position of the i-th osprey is defined as

(19) $$X_{i}=\left[x_{i,1},x_{i,2},\ldots ,x_{i,m}\right],x_{i,j}\in R$$

where $X_{i}$ denotes the continuous position vector of the i-th osprey, $x_{i,j}$ is its position in the j-th dimension, and m is the number of original features. The corresponding binary feature selection vector is defined as

(20) $$B_{i}=\left[B_{i,1},B_{i,2},\ldots ,B_{i,m}\right],B_{i,j}\in \left\{0,1\right\}$$

$B_{i}$ represents the selected feature subset of the i-th osprey. If $B_{i,j}=1$, the j-th feature is selected; otherwise, it is discarded. During optimization, ABOOA first updates the continuous position $X_{i}$, then converts it into the binary vector $B_{i}$ via a binary mapping, and finally determines whether the new solution should be accepted according to the fitness of $B_{i}$.

To improve the discriminative capability of the Sigmoid-based binary mapping, the continuous search space is defined over a symmetric interval with $lb_{j}=-6$ and $ub_{j}=6$. This setting allows the continuous variable $x_{i,j}$ to span both positive and negative regions. Consequently, the Sigmoid output is not biased toward values greater than 0.5, which would otherwise lead to selecting an excessive number of features when the search space is limited to [0, 1]. The symmetric search boundary provides better control over feature selection probabilities and facilitates the generation of more compact feature subsets.

#### 3.3.1. Circle Chaotic Initialization Strategy

The original OOA initializes the population randomly. Although simple, random initialization may produce unevenly distributed individuals, limiting the coverage of promising search regions and reducing the efficiency of subsequent optimization. To address this issue, ABOOA employs a circle chaotic mapping to generate initial continuous positions, thereby improving population diversity and search-space coverage.

The circle chaotic map is defined as

(21) $$z_{k+1}=\mathrm{mod}(z_{k}+b-\frac{a}{2\pi }sin(2\pi z_{k}),1)$$

where $z_{k}$ is the chaotic variable generated at the k-th iteration, a and b are control parameters, and mod(⋅) denotes the modulo operation. In this study, a=0.5 and b=0.2 are adopted to improve the ergodicity of the chaotic sequence over the interval [0, 1]. To avoid sequence degeneration, the initial value $z_{0}$ is randomly selected from the open interval (0, 1).

Based on the generated chaotic sequence, the initial continuous position of the i-th osprey in the j-th dimension is computed as

(22) $$x_{i,j}^{0}=lb_{j}+z_{i,j}\cdot (ub_{j}-lb_{j}),i=1,2,\ldots ,N;j=1,2,\ldots ,m$$

where $x_{i,j}^{0}$ denotes the initial position, $lb_{j}$ and $ub_{j}$ are the lower and upper bounds of the j-th dimension, and $z_{i,j}\in [0,1]$ is the chaotic variable generated by the circle map. Since the search interval is set to [−6, 6], the initialized positions are distributed symmetrically around zero, providing a suitable basis for the subsequent Sigmoid-based binary mapping.

Because feature selection is a binary optimization problem, the initialized continuous positions must be converted into feature selection probabilities. The Sigmoid function is adopted for this purpose.

(23) $$S(x_{i,j}^{0})=\frac{1}{1+exp(-x_{i,j}^{0})}$$

$S(x_{i,j}^{0})$ denotes the initial probability of selecting the j-th feature for the i-th osprey. According to this probability, the initial binary feature selection state is determined as

(24) $$B_{i,j}^{0}=\left\{\begin{array}{cc} 1, & \mathrm{if}\;\mathrm{rand}<S(x_{i,j}^{0})\\ 0, & otherwise \end{array}\right.$$

where rand∈[0,1] is a uniformly distributed random number, and $B_{i,j}^{0}$ denotes the initial binary state of the j-th feature. By introducing circle chaotic initialization, ABOOA generates more diverse candidate feature subsets during the early search stage, increases population diversity, and provides higher-quality initial solutions for subsequent global exploration and local exploitation.

#### 3.3.2. Adaptive Search Strategy

The original OOA uses relatively fixed position-update rules during both global exploration and local exploitation. As a result, the search intensity cannot be adjusted effectively as the iteration proceeds. For high-dimensional feature selection, larger search steps are needed early to expand the search space and reduce the risk of premature convergence. In later iterations, the perturbation should gradually decrease, allowing the population to refine promising regions more precisely. To achieve this transition, ABOOA introduces an adaptive search factor that varies with each iteration.

Let T denote the maximum number of iterations and t the current iteration, where t=1,2,…,T. The adaptive search factor is defined as

(25) $$\omega (t)=\omega_{min}+(\omega_{max}-\omega_{min})\cdot {\left(1-\frac{t}{T}\right)}^{2}$$

$\omega_{max}$ and $\omega_{min}$ represent the maximum and minimum values of the search factor, respectively. As the iteration number increases, ω(t) decreases nonlinearly. A larger value in the early stage promotes broad exploration and helps maintain population diversity. In the later stage, the smaller factor reduces excessive movement and improves the stability and convergence accuracy of local exploitation.

During global exploration, ABOOA constructs a candidate prey set based on the fitness values of binary feature subsets. For the i-th osprey, the candidate prey set is defined as

(26) $$S_{i}=\{X_{k}|F(B_{k})<F(B_{i}),k\in \{1,2,\ldots ,N\},k\neq i\}$$

$S_{i}$ denotes the candidate prey set of the i-th osprey, and $F(B_{i})$ is the fitness value of its binary feature subset $B_{i}$. It should be noted that the optimization target of ABOOA is the feature subset instead of the continuous position itself. Therefore, candidate prey are selected based on $F(B_{k})<F(B_{i})$ rather than $F(X_{k})<F(X_{i})$. If $S_{i}$ is empty, an individual other than $X_{i}$ is randomly selected from the population. One member of $S_{i}$ is then chosen as the prey position of the i-th osprey and denoted by $FP_{i}$.

Based on the global exploration rule of the original OOA, ABOOA introduces the adaptive search factor into the movement toward the prey position.

(27) $$x_{i,j}^{\mathrm{new},P1}=x_{i,j}+\omega \left(t\right)\cdot r_{i,j}\cdot \left(FP_{i,j}-I_{i,j}\cdot x_{i,j}\right),i=1,2,\ldots ,N;j=1,2,\ldots ,m$$

where $x_{i,j}^{\mathrm{new},P1}$ denotes the updated continuous position of the i-th osprey in the j-th dimension during global exploration, $x_{i,j}$ is the current position, and $FP_{i,j}$ is the corresponding component of the selected prey position $FP_{i}$. In addition, $r_{i,j}\in [0,1]$ is a random number, and $I_{i,j}\in \{1,2\}$ is a randomly generated integer. Compared with the original global exploration rule, Equation (27) includes ω(t) to regulate the movement amplitude. This process allows broader exploration in the early stage and progressively smaller updates near convergence, thereby improving search stability.

During local exploitation, the adaptive search factor is further introduced into the local perturbation term. The updated position is calculated as

(28) $$x_{i,j}^{\mathrm{new},P2}=x_{i,j}+\omega (t)\cdot \frac{lb_{j}+r_{i,j}\cdot (ub_{j}-lb_{j})}{t},i=1,2,\ldots ,N;j=1,2,\ldots ,m$$

where $x_{i,j}^{\mathrm{new},P2}$ is the updated continuous position of the i-th osprey in the j-th dimension during local exploitation. The lower and upper bounds of the j-th dimension are denoted by $lb_{j}$ and $ub_{j}$, respectively. The random variable $r_{i,j}$ lies in [0, 1], and t is the current iteration number. As shown in Equation (28), the perturbation amplitude is jointly controlled by ω(t) and 1/t. Both terms decrease as the iteration proceeds. Therefore, the search gradually shifts from broad exploration to fine-grained refinement, improving convergence precision in the later stage.

To prevent the updated continuous position from exceeding the feasible search range, a boundary constraint is applied after each update.

(29) $$x_{i,j}^{\mathrm{new}}=min\left(max\left(x_{i,j}^{\mathrm{new}},lb_{j}\right),ub_{j}\right)$$

$x_{i,j}^{\mathrm{new}}$ is obtained from either global exploration or local exploitation. This operation ensures that each coordinate remains within $\left[lb_{j},ub_{j}\right]$, preventing invalid positions from affecting subsequent binary mapping and fitness evaluation.

After the continuous position is updated, the new vector $X_{i}^{\mathrm{new}}$ is converted into a binary feature selection vector $B_{i}^{\mathrm{new}}$ through the adaptive binary mapping mechanism. Greedy selection is then performed according to the fitness of the resulting feature subset.

(30) $$\left(X_{i},B_{i}\right)\leftarrow \left\{\begin{array}{cc} \left(X_{i}^{\mathrm{new}},B_{i}^{\mathrm{new}}\right), & \mathrm{if}\;F\left(B_{i}^{\mathrm{new}}\right)<F\left(B_{i}\right)\\ \left(X_{i},B_{i}\right), & \mathrm{otherwise} \end{array}\right.$$

Here, $X_{i}^{\mathrm{new}}$ denotes the continuous position generated during exploration or exploitation stage, whereas $B_{i}^{\mathrm{new}}$ denotes the binary vector obtained from $X_{i}^{\mathrm{new}}$. If the new feature subset achieves a better fitness value, both the continuous position and its binary representation are accepted. Otherwise, the original pair is retained. Unlike the original OOA, which updates only continuous positions, ABOOA maintains $X_{i}$ and $B_{i}$ simultaneously. This design preserves consistency between the continuous search space and the binary feature selection space.

#### 3.3.3. Adaptive Binary Mapping Mechanism

Feature selection is essentially a discrete binary optimization problem, whereas OOA generates continuous-valued positions. Therefore, converting continuous search positions into binary feature selection states is a key step in applying ABOOA to feature selection. A fixed Sigmoid function keeps the same mapping intensity throughout the optimization process. This procedure may reduce randomness in the early stage or lead to unstable feature states near convergence. To address this issue, ABOOA introduces an adaptive binary mapping mechanism. A time-varying factor dynamically regulates the selection probability, enabling stronger exploration early on and more stable binary decisions later.

The continuous position $x_{i,j}$ is first transformed into the probability of selecting the j-th feature:

(31) $$P_{i,j}(t)=\frac{1}{1+exp(-\lambda (t)\cdot x_{i,j})}$$

where $P_{i,j}(t)$ denotes the probability that the i-th osprey selects the j-th feature at iteration t. The adaptive mapping factor λ(t) controls the steepness of the Sigmoid function. Since the continuous search interval is symmetric around zero, $x_{i,j}$ can take both positive and negative values. A larger $x_{i,j}$ increases the selection probability, whereas a smaller $x_{i,j}$ increases the likelihood that the feature will be removed.

The adaptive mapping factor is defined as

(32) $$\lambda (t)=\lambda_{min}+(\lambda_{max}-\lambda_{min})\cdot \frac{t}{T}$$

$\lambda_{min}$ and $\lambda_{max}$ are the minimum and maximum values of the mapping factor, respectively. At the beginning of optimization, λ(t) is relatively small, resulting in a smoother Sigmoid curve and more random feature selection probabilities. This process helps maintain population diversity. As the iteration proceeds, λ(t) increases and the curve becomes steeper. Continuous positions are therefore more likely to map to stable binary states, improving the consistency of the selected feature subsets.

According to $P_{i,j}(t)$, the binary state of the j-th feature is determined as

(33) $$B_{i,j}=\left\{\begin{array}{cc} 1, & \mathrm{if}\;\mathrm{rand}<P_{i,j}(t)\\ 0, & otherwise \end{array}\right.$$

where rand∈[0,1] is a uniformly distributed random number. If $B_{i,j}=1$, the j-th feature is retained; otherwise, it is discarded. This mapping mechanism links continuous optimization with discrete feature selection. Each osprey can update its position in the continuous space while representing a specific feature subset in binary form.

To evaluate the quality of a candidate feature subset, ABOOA uses a fitness function that balances classification performance and subset size.

(34) $$F(B_{i})=\alpha \cdot (1-Acc_{i})+(1-\alpha )\cdot \frac{|B_{i}|}{m}$$

where $Acc_{i}$ denotes the validation accuracy obtained using the i-th binary feature subset $B_{i}$, $|B_{i}|$ is the number of selected features, and m represents the total number of original features. The parameter α∈[0,1] controls the trade-off between classification performance and subset size. In the experiments, α is set to 0.9 to place greater emphasis on classification performance while retaining a penalty on the number of selected features. The first term penalizes classification errors, while the second limits the number of retained features. By minimizing Equation (34), ABOOA removes redundant dimensions while preserving detection performance, yielding a compact, discriminative feature subset. To provide an intuitive illustration of the binary mapping and fitness evaluation process, consider a simplified example with four candidate features. At a given iteration, suppose that the scaled position values entering the Sigmoid function in Equation (31) are $\left[\lambda \left(t\right)x_{i,1},\;\lambda \left(t\right)x_{i,2},\;\lambda \left(t\right)x_{i,3},\;\lambda (t)x_{i,4}\right]=\left[-1.2,\;0.8,\;2.0,\;-0.5\right]$. According to Equation (31), the corresponding feature-selection probabilities are $\left[P_{i,1}\left(t\right),P_{i,2}\left(t\right),P_{i,3}\left(t\right),P_{i,4}(t)\right]=\left[0.23,\;0.69,\;0.88,\;0.38\right]$. Suppose that the uniformly sampled random values for the four features are $\left[0.40,\;0.50,\;0.60,\;0.70\right]$. According to Equation (33), the resulting binary feature-selection vector is $B_{i}=\left[0,\;1,\;1,\;0\right]$, indicating that the second and third features are retained. If the selected feature subset achieves a validation accuracy of 0.92, then $\left|B_{i}\right|=2$. With α=0.9, the fitness value defined in Equation (34) is calculated as $F(B_{i})=0.9\left(1-0.92\right)+0.1\times \frac{2}{4}=0.122$. This example illustrates how the adaptive Sigmoid mapping converts continuous search information into feature-selection probabilities, how these probabilities are further transformed into a discrete feature subset, and how classification performance and feature compactness are jointly considered during fitness evaluation.

In summary, ABOOA improves the original OOA in three aspects. First, circle chaotic mapping enhances the diversity of the initial population and expands its coverage of the search space. Second, the adaptive search factor dynamically regulates the search intensity during global exploration and local exploitation. This process enables the algorithm to emphasize broad exploration in early iterations and gradually shift toward local refinement. Third, the adaptive binary mapping mechanism converts continuous positions into binary feature selection vectors, allowing OOA to handle high-dimensional discrete optimization tasks effectively. These improvements make ABOOA better suited to multimodal fake news detection, where features are often high-dimensional, redundant, and strongly correlated. The selected subsets provide a more compact, stable, and discriminative basis for subsequent cross-modal relation modeling and news authenticity classification.

### 3.4. Cross-Modal Relation Disentanglement Module (CRDM)

After ABOOA feature selection, redundant dimensions in both image and text features are removed, leaving representations that are more relevant to the downstream task. However, feature selection alone cannot reveal the semantic relationships between the two modalities. In multimodal fake news detection, the relationship between images and text is more complex than simple similarity or dissimilarity. It usually involves semantic consistency, discrepancy, and complementarity simultaneously. Conventional fusion methods, such as feature concatenation, weighted summation, or standard attention mechanisms, tend to mix these heterogeneous relationships into a single representation space, making it difficult to distinguish semantic consistency, modality-specific characteristics, and cross-modal complementary information.

To address this issue, we propose the Cross-modal Relation Disentanglement Module (CRDM). Using the image and text features selected by ABOOA as inputs, CRDM explicitly decomposes cross-modal relationships into three semantic components: shared, discrepant, and complementary information. Shared information captures semantic evidence that is consistent across the two modalities. Discrepant information preserves modality-specific cues and semantic inconsistencies between images and text. Complementary information models the additional semantics generated through cross-modal interaction. Instead of performing another fusion operation directly, CRDM first establishes a structured representation of different semantic relationships. Each type of cross-modal evidence is modeled independently before being adaptively integrated by the subsequent fusion module.

Let $F_{v}$ and $F_{t}$ denote the image and text features selected by ABOOA, respectively. To reduce the distribution gap between modalities, they are first projected into a common latent space.

(35) $$H_{v}=\phi (W_{v}\cdot F_{v}+b_{v})$$

(36) $$H_{t}=\phi (W_{t}\cdot F_{t}+b_{t})$$

$H_{v}$ and $H_{t}$ denote the latent representations of the image and text modalities, respectively. $W_{v}$ and $W_{t}$ are learnable weight matrices, $b_{v}$ and $b_{t}$ are bias vectors, and ϕ(⋅) is a nonlinear activation function. After projection, both modalities share the same feature dimension, providing a common representation space for subsequent relation disentanglement.

#### 3.4.1. Shared Information Modeling

The purpose of shared information modeling is to extract semantic content that is consistent across images and text. In real news, the objects, scenes, or emotional tendencies described in the text are often supported by the accompanying image. In contrast, fake news frequently exhibits image–text mismatches, weak semantic correlations, or partial inconsistencies. Therefore, shared information provides important evidence for evaluating semantic consistency between the two modalities.

A cross-modal interaction vector is first constructed as

(37) $$Q_{c}=[H_{v};H_{t};H_{v}⊙H_{t};|H_{v}-H_{t}|]$$

where $Q_{c}$ is the input for shared information modeling, ⊙ denotes element-wise multiplication, [;] represents feature concatenation, and $|H_{v}-H_{t}|$ is the absolute difference between the image and text representations. The term $H_{v}⊙H_{t}$ captures consistent responses across corresponding feature dimensions, while $|H_{v}-H_{t}|$ measures their semantic discrepancy.

Based on $Q_{c}$, the shared-information attention weight is computed as

(38) $$A_{c}=\mathrm{sigmoid}(W_{c}\cdot Q_{c}+b_{c})$$

where $W_{c}$ and $b_{c}$ are learnable parameters. sigmoid(⋅) constrains the attention weights to the interval [0, 1].

The shared representation is then obtained by

(39) $$C=A_{c}⊙\left[\frac{H_{v}+H_{t}}{2}\right]$$

C denotes the shared information between the image and text modalities. The average semantic representation of the two modalities serves as the basic representation, while the attention weight $A_{c}$ emphasizes dimensions with strong semantic agreement. Features exhibiting high cross-modal consistency are preserved, whereas weakly correlated responses are suppressed.

#### 3.4.2. Discrepant Information Modeling

Although images and text may describe the same event, they often emphasize different aspects. Text is better suited to expressing event background, opinions, and emotional tendencies, whereas images directly present scenes, people, and visual details. In fake news detection, such discrepancies should not be regarded as noise. Instead, image–text inconsistency, misleading visual content, and exaggerated textual descriptions often provide valuable cues for authenticity prediction. Therefore, in addition to shared information, modality-specific information should also be retained.

Using the shared representation C as a semantic reference, the residual information of each modality is extracted as follows:

(40) $$D_{v}=H_{v}-C$$

(41) $$D_{t}=H_{t}-C$$

$D_{v}$ and $D_{t}$ denote the residual representations of the image and text modalities, respectively. By removing the shared semantics, the residual representations emphasize modality-specific characteristics and semantic discrepancies while reducing redundant information.

To adaptively measure the importance of these two residual representations, a discrepancy gate is introduced.

(42) $$G_{d}=\mathrm{sigmoid}(W_{d}\cdot [D_{v};D_{t};|D_{v}-D_{t}|]+b_{d})$$

where $G_{d}$ denotes the gating weight for discrepant information, $|D_{v}-D_{t}|$ measures the difference between the image and text residuals, and $W_{d}$ and $b_{d}$ are learnable parameters.

The final discrepant representation is computed as

(43) $$D=G_{d}⊙D_{v}+(1-G_{d})⊙D_{t}$$

where D represents the integrated discrepant information. The gating mechanism dynamically adjusts the contributions of image- and text-specific residuals for each sample, enabling the model to capture image–text inconsistency, modality-specific evidence, and potential semantic conflicts more effectively.

#### 3.4.3. Complementary Information Modeling

Shared information emphasizes semantic consistency between images and text, whereas discrepant information captures modality-specific characteristics and semantic inconsistency. In contrast, complementary information focuses on the additional semantics generated through cross-modal interaction. In some cases, neither the image nor the text alone provides sufficient evidence for authenticity prediction, while their combination reveals more informative semantic cues. For example, a textual description may appear reasonable by itself, yet become suspicious when compared with the corresponding image due to inconsistencies in objects, locations, or context. Likewise, an image without an obvious semantic tendency may become highly informative when interpreted together with the accompanying text.

To capture such complementary semantics, a bidirectional interaction mechanism is designed. The image representation is first enhanced under the guidance of textual information.

(44) $$M_{v}=tanh(W_{mv}\cdot [H_{v};H_{t};C;D]+b_{mv})$$

where $M_{v}$ denotes the text-guided complementary representation of the image modality.

Similarly, textual semantics are enhanced using image information.

(45) $$M_{t}=tanh(W_{mt}\cdot [H_{t};H_{v};C;D]+b_{mt})$$

where $M_{t}$ denotes the image-guided complementary representation of the text modality. $W_{mv}$, $W_{mt}$, $b_{mv}$, and $b_{mt}$ are learnable parameters, and tanh(⋅) introduces nonlinear interactions. Unlike directly modeling the interaction between $H_{v}$ and $H_{t}$, Equations (44) and (45) additionally incorporate the shared representation C and the discrepant representation D. Consequently, complementary information is learned based on both semantic consistency and semantic discrepancy rather than simply reusing the original modality representations.

The complementary gating weight is then computed as

(46) $$G_{m}=\mathrm{sigmoid}(W_{m}\cdot [M_{v};M_{t}]+b_{m})$$

Moreover, the final complementary representation is obtained by

(47) $$M=G_{m}⊙M_{v}+(1-G_{m})⊙M_{t}$$

where $G_{m}$ denotes the gating weight, and M represents the final complementary information. The gating mechanism adaptively balances the contributions of the text-guided image representation and the image-guided text representation for each sample, enabling the model to extract more discriminative cross-modal complementary cues.

#### 3.4.4. Relation Disentanglement Constraint

Generating shared, discrepant, and complementary representations does not ensure that they capture distinct semantic information. If C, D, and M remain highly similar in the latent space, the subsequent fusion module will still receive redundant information, reducing the effectiveness of relation disentanglement. To encourage semantic separation among the three representations, a relation disentanglement constraint is introduced.

Assume that a mini-batch contains n training samples. For the q-th sample, the corresponding shared, discrepant, and complementary representations are denoted by $C_{q}$, $D_{q}$, and $M_{q}$, respectively. The relation disentanglement loss is defined as

(48) $$L_{dec}=\frac{1}{n}\sum_{q=1}^{n}\left[{cos}^{2}\left(C_{q},D_{q}\right)+{cos}^{2}\left(C_{q},M_{q}\right)+{cos}^{2}\left(D_{q},M_{q}\right)\right]$$

where $L_{dec}$ denotes the disentanglement loss and cos(⋅) represents cosine similarity. Minimizing this loss reduces semantic redundancy among the three representations, encouraging each branch to capture a distinct type of cross-modal evidence.

Rather than using mutual information minimization or orthogonality constraints, this study introduces a relation disentanglement loss to regulate the learned representations. Specifically, the pairwise cosine similarities among the shared, discrepant, and complementary representations are minimized during training. In this way, semantic overlap among different relation representations is reduced, allowing each branch to focus on distinct cross-modal information and promoting better separation of the corresponding latent representations.

To prevent disentanglement from weakening classification performance, an auxiliary classification objective is introduced. The three relation representations are first concatenated.

(49) R=[C;D;M]

Furthermore, the auxiliary prediction is generated by

(50) $${\hat{y}}_{r}=\mathrm{softmax}(W_{r}\cdot R+b_{r})$$

The auxiliary classification loss is defined as

(51) $$L_{aux}=-\frac{1}{n}\sum_{q=1}^{n}\sum_{c=1}^{K}y_{q,c}\cdot log({\hat{y}}_{r,q,c}+\varepsilon )$$

where K is the number of categories, $y_{q,c}$ denotes the ground-truth label of the q-th sample for class c, ${\hat{y}}_{r,q,c}$ is the predicted probability of the auxiliary classifier, and ε is a small constant introduced to ensure numerical stability. This objective encourages the disentangled representations to remain discriminative for fake news detection while preserving semantic separation.

The overall optimization objective of CRDM is formulated as follows:

(52) $$L_{CRDM}=L_{aux}+\beta \cdot L_{dec}$$

where β is the weighting factor that balances classification performance and relation disentanglement.

In summary, CRDM further decomposes the selected multimodal features into three semantic components: shared information C, discrepant information D, and complementary information M. Shared information models semantic consistency between images and text, discrepant information captures modality-specific characteristics and semantic inconsistency, while complementary information represents the additional semantics generated through cross-modal interaction. Unlike conventional fusion methods that directly combine multimodal features, CRDM first establishes a task-oriented relation decomposition structure, allowing different types of cross-modal evidence to be modeled independently before fusion. Furthermore, the relation disentanglement constraint reduces redundancy among the three representations, and the auxiliary classification objective preserves their discriminative capability for fake news detection. As a result, CRDM produces cross-modal representations with clearer semantic structures and better-defined boundaries, providing reliable inputs to the subsequent Adaptive Semantic Fusion Module (ASFM).

### 3.5. Adaptive Semantic Fusion and Classification (ASFM)

After the CRDM, the model obtains three types of cross-modal relation representations: shared information C, discrepant information D, and complementary information M. These representations characterize semantic consistency, modality discrepancy, and cross-modal complementarity, respectively. However, their contributions vary across different samples. For news with highly consistent image–text pairs, shared information is usually the most informative. When a semantic mismatch or conflict exists, discrepant information provides stronger evidence for authenticity prediction. For samples with insufficient evidence from a single modality, complementary information supplies additional semantic cues. Therefore, assigning fixed fusion weights to C, D, and M cannot effectively accommodate the varying cross-modal relationships among different samples.

To address this issue, an Adaptive Semantic Fusion Module (ASFM) is proposed to dynamically integrate the three relation representations produced by CRDM. Given inputs C, D, and M, ASFM generates sample-specific fusion weights based on the current cross-modal relationship and produces the final multimodal semantic representation. Unlike simple concatenation or average fusion, ASFM adaptively adjusts the contribution of each relation type before classification.

Based on the three relation representations, the unnormalized importance scores are computed as follows:

(53) $$s=W_{a}\cdot R+b_{a},s=[s_{c},s_{d},s_{m}]$$

Here $s\in R^{3}$. The elements $s_{c}$, $s_{d}$, and $s_{m}$ represent the scores of the shared, discrepant, and complementary information, respectively. $W_{a}$ and $b_{a}$ are learnable parameters. These scores measure the relative importance of the three relation types for the current sample.

To obtain comparable weights whose sum equals one, the Softmax function is applied.

(54) $$\left[\alpha_{c},\alpha_{d},\alpha_{m}]=\mathrm{softmax}(s\right)$$

where $\alpha_{c}$, $\alpha_{d}$, and $\alpha_{m}$ denote the adaptive fusion weights of C, D, and M, respectively, satisfying.

(55) $$\alpha_{c}+\alpha_{d}+\alpha_{m}=1$$

Using these adaptive weights, the final multimodal semantic representation is calculated as

(56) $$Z=\alpha_{c}\cdot C+\alpha_{d}\cdot D+\alpha_{m}\cdot M$$

Z is the fused representation generated by ASFM. The weights $\alpha_{c}$, $\alpha_{d}$, and $\alpha_{m}$ are sample-level scalars that control the contributions of the three relation representations. Consequently, the model dynamically emphasizes the most informative semantic evidence instead of combining them with fixed coefficients. When semantic consistency dominates the prediction, $\alpha_{c}$ becomes larger. If image–text inconsistency provides stronger evidence, $\alpha_{d}$ receives a higher weight. Likewise, when cross-modal interaction provides additional semantic evidence, $\alpha_{m}$ becomes larger. For example, consider a sample for which ASFM assigns the following weights to the shared, discrepant, and complementary representations: $\alpha_{c}=0.60$, $\alpha_{d}=0.30$, $\alpha_{m}=0.10$. The final fused representation is therefore calculated as Z=0.60·C+0.30·D+0.10·M. In this example, the shared representation receives the largest weight, indicating that cross-modal semantic consistency provides the dominant evidence for the current sample. For a sample exhibiting stronger image–text inconsistency, a larger weight may instead be assigned to the discrepant representation. This example illustrates how ASFM adaptively adjusts the contributions of different cross-modal relations according to sample-specific semantic characteristics.

To further improve the representation capability, the fused feature is transformed by a nonlinear projection.

(57) $$Z_{f}=\phi (W_{f}\cdot Z+b_{f})$$

where $Z_{f}$ denotes the final feature for classification, $W_{f}$ and $b_{f}$ are learnable parameters, and ϕ(⋅) is a nonlinear activation function. This transformation further refines the fused representation, making it more suitable for downstream classification.

In the prediction stage, $Z_{f}$ is fed into the classifier to estimate the probability distribution over all categories.

(58) $$\hat{y}=\mathrm{softmax}(W_{o}\cdot Z_{f}+b_{o})$$

where $\hat{y}$ is the final prediction, and $W_{o}$ and $b_{o}$ are the classifier parameters. For binary fake news detection, $\hat{y}$ represents the probabilities that a sample belongs to the real-news or fake-news category. The predicted label is determined by

(59) $$y_{pred}=arg\;max(\hat{y})$$

To optimize the final prediction, the cross-entropy loss is adopted as the primary classification objective. Assume that a mini-batch contains n samples and the number of categories is K. Let $y_{q,c}$ denote the ground-truth label of the q-th sample for class c, and ${\hat{y}}_{q,c}$ be the corresponding predicted probability. The classification loss is defined as

(60) $$L_{cls}=-\frac{1}{n}\sum_{q=1}^{n}\sum_{c=1}^{K}y_{q,c}\cdot log({\hat{y}}_{q,c}+\varepsilon )$$

where ε is a small constant introduced to ensure numerical stability, the loss $L_{cls}$ directly supervises the final prediction and aligns the fused representation $Z_{f}$ with the objective of fake news detection.

Together with the relation disentanglement loss and the auxiliary classification loss introduced in CRDM, the overall optimization objective is formulated as

(61) $$L_{total}=L_{cls}+\mu \cdot L_{CRDM}$$

where μ is the weighting coefficient of the CRDM objective. Substituting the definition of $L_{CRDM}$, the overall optimization objective is formulated as

(62) $$L_{total}=L_{cls}+\mu \cdot (L_{aux}+\beta \cdot L_{dec})$$

where $L_{cls}$ optimizes the final classification performance, $L_{aux}$ preserves the discriminative capability of the disentangled relation representations, and $L_{dec}$ reduces semantic redundancy among the shared, discrepant, and complementary information. By jointly optimizing these objectives, the model simultaneously improves classification accuracy and maintains a well-structured cross-modal representation space.

In summary, ASFM adaptively learns fusion weights for the shared, discrepant, and complementary information based on each sample’s cross-modal relationship and generates the final feature representation $Z_{f}$. Unlike fixed fusion strategies, ASFM flexibly adjusts the contribution of each relation type to accommodate diverse semantic patterns across different samples. The classifier then predicts the authenticity of the news based on $Z_{f}$. Together with ABOOA and CRDM, ASFM forms an integrated sequential framework that combines feature selection, relation disentanglement, adaptive semantic fusion, and classification for multimodal fake news detection. Specifically, CRDM preserves semantic inconsistency and modality-specific cues between text and images in the discrepant representation D, allowing cross-modal conflicts to enter the subsequent fusion as a distinct relation. In ASFM, the shared, discrepant, and complementary representations C, D, and M are associated with adaptive weights $\alpha_{c}$, $\alpha_{d}$, and $\alpha_{m}$, respectively. These weights are learned according to the cross-modal characteristics of each sample. Thus, the contribution of discrepant information is adaptively controlled by $\alpha_{d}$, rather than being represented merely as low cross-modal similarity. In this way, the final decision incorporates semantic consistency, discrepancy, and complementary evidence simultaneously.

## 4. Experimental Results and Analysis

### 4.1. Experimental Settings

#### 4.1.1. Dataset

The dataset adopted in this research is gathered from three reliable Chinese information platforms: Sina News, Science China, and the China Internet Joint Rumor Refutation Platform. It includes authentic news articles, online misinformation, and scientific explanations to clarify rumors. The number of samples obtained from each source is presented in Table 1. To maintain data representativeness across different sources and avoid potential information leakage, a stratified random sampling approach is employed to partition the dataset. Specifically, 70% of the samples are allocated to model training, 15% to validation, and the remaining 15% to testing. The validation subset is used for hyperparameter optimization and model selection, while the test subset is used solely to assess the final performance of the proposed model.

It should be noted that the text and image counts in Table 1 refer to the raw data collected from each source and are therefore not fully matched. Before multimodal feature extraction, only valid text–image pairs are retained, while samples with missing or unmatched modalities are removed.

#### 4.1.2. Evaluation Metrics

To comprehensively evaluate the classification performance, four widely used metrics are adopted: Accuracy (Acc), Precision (Pre), Recall (Rec), and F1-score (F1). Accuracy measures the model’s overall prediction accuracy. Precision indicates the proportion of correctly predicted positive samples among all predicted positives. Recall measures the model’s ability to identify actual positive samples. F1-score is the harmonic mean of Precision and Recall, providing a balanced assessment of classification performance.

#### 4.1.3. Experimental Hyperparameter Settings

To improve training stability and generalization, Early Stopping is employed based on the validation performance. Training is terminated when the validation performance fails to improve for several consecutive epochs. In addition, Label Smoothing, Dropout, and gradient clipping are applied to further enhance convergence stability. The main hyperparameter settings are listed in Table 2.

### 4.2. Comparison with Multimodal Baseline Models

To comprehensively evaluate the performance of the proposed ABOOA-CDF model for multimodal fake news detection, six representative baseline models are selected, including MCAN, EANN, SpotFake+, BCMF, SAFE, and MCNN. These methods represent different technical paradigms, such as cross-modal attention, adversarial learning, deep multimodal fusion, and convolutional neural networks. To reduce the influence of data partitioning and model initialization on a single experimental run, five-fold cross-validation is adopted. Accuracy, Precision, Recall, and F1-score are reported as mean ± standard deviation (Mean ± SD). All methods use the same input features, evaluation metrics, and training settings to ensure a fair comparison. The results are presented in Table 3, with the best value for each metric highlighted in bold.

MCAN [38]: MCAN employs a multi-layer cross-modal co-attention mechanism to model interactions between textual and visual information. This design enhances cross-modal feature interaction but introduces a relatively complex architecture that incurs a higher computational cost.

EANN: EANN adopts an event-adversarial learning framework to learn event-invariant representations, thereby improving generalization across different events. However, its ability to model fine-grained semantic relationships between images and text remains limited.

SpotFake+: SpotFake+ performs multimodal fusion via textual encoding and visual feature extraction, yielding effective joint image–text representations. Nevertheless, it is less effective at capturing deep semantic interactions across modalities.

BCMF [39]: BCMF models textual and visual information using a dual-branch collaborative fusion architecture, thereby enhancing multimodal feature representation. However, it does not explicitly distinguish between shared and modality-specific information.

SAFE: SAFE focuses on semantic consistency between images and text for detecting fake news. Although it performs well on samples with semantic conflicts, its effectiveness may decline when forged content exhibits strong image–text semantic consistency.

MCNN: A multi-channel convolutional neural network that extracts multimodal features and performs joint classification. While it provides effective feature extraction, its cross-modal fusion strategy is relatively simple.

The relatively strong performance of the baseline models may be attributed to the informative visual content and semantic relationships between text and images, which provide useful cues for multimodal fake news detection. To ensure a fair comparison, all methods use the same input features and training settings. Since class distribution may affect Accuracy, stratified sampling is applied to maintain consistent class proportions across different data subsets. Precision, Recall, and F1-score are also considered to provide a more comprehensive evaluation. As shown in Table 3, although the models achieve relatively high Accuracy, noticeable differences remain in the other three metrics. Therefore, high Accuracy alone does not fully reflect the ability to distinguish between different classes, nor can the overall performance be attributed to class distribution. ABOOA-CDF achieves the highest mean values for all four evaluation metrics. Its Accuracy reaches 96.93 ± 0.56%, exceeding the strongest baseline, SAFE, by 1.02%. The model obtains a Precision of 93.00 ± 1.97%, an improvement of 1.06% over the best baseline result for this metric. Its Recall is 93.15 ± 0.95%, which is 1.39% higher than SAFE’s. For F1-score, ABOOA-CDF achieves 93.07 ± 1.23%, surpassing SAFE by 2.24%. Overall, the proposed model maintains high Precision while improving Recall, leading to a better balance between the two metrics and stronger overall detection performance.

The baseline methods show different performance characteristics. MCAN and MCNN achieve Precision values of 91.94 ± 1.54% and 91.52 ± 1.91%, respectively, and Recall values of 86.39 ± 0.84% and 87.59 ± 0.89%, respectively. This suggests that their ability to identify positive samples remains relatively limited despite their high Precision. EANN and SpotFake+ achieve similar Accuracy, while SpotFake+ obtains a higher F1-score of 90.15 ± 0.65%. In contrast, BCMF and SAFE perform better in Recall, reaching 90.93 ± 1.37% and 91.76 ± 1.44%, respectively, but show relatively lower Precision. Overall, the baseline methods exhibit different trade-offs between Precision and Recall, making it difficult to achieve balanced performance across all evaluation metrics.

ABOOA-CDF addresses these limitations through feature optimization, cross-modal relation modeling, and adaptive semantic fusion. First, ABOOA selects informative textual and visual features while reducing the influence of redundant information. The cross-modal relation disentanglement module then separates the relationships between text and images into shared, discrepant, and complementary representations, allowing different types of semantic information to be modeled individually. Based on these representations, ASFM dynamically adjusts its contributions based on each sample’s characteristics and integrates them into the final multimodal representation. This design provides a better balance between Precision and Recall while improving overall detection performance. The results across the five folds further demonstrate the effectiveness and stability of ABOOA-CDF.

### 4.3. Component Ablation Experiment

To further investigate the contribution of each module and loss function in ABOOA-CDF, a series of ablation experiments is conducted while keeping all other experimental settings unchanged. Specifically, the shared representation module (w/o C), the discrepant representation module (w/o D), the complementary representation module (w/o M), and the Adaptive Semantic Fusion Module (w/o ASFM) are removed individually. In addition, the auxiliary classification loss (w/o Aux) and the relation disentanglement loss (w/o Dec) are excluded separately to evaluate their respective contributions. The experimental results are summarized in Table 4. The bold indicates the best performance.

As shown in Table 4, removing any module or loss function leads to a decline in performance, indicating that each component of ABOOA-CDF contributes positively to multimodal fake news detection. When the shared representation module (w/o C) is removed, all evaluation metrics decrease, demonstrating that shared information effectively captures semantic consistency between text and images and provides an essential basis for subsequent cross-modal fusion. Similarly, removing the discrepant representation module (w/o D) also degrades performance, indicating that preserving modality-specific information helps distinguish complex samples with semantic inconsistencies.

Removing the complementary representation module (w/o M) results in lower Accuracy and F1-score, suggesting that complementary information enriches multimodal feature representations and improves the detection of different types of fake news. A more noticeable performance drop is observed when the Adaptive Semantic Fusion Module (w/o ASFM) is removed. This result indicates that fixed fusion strategies cannot fully exploit shared, discrepant, and complementary information. In contrast, the adaptive fusion mechanism dynamically adjusts the contributions of each semantic representation based on the characteristics of individual samples, resulting in more informative multimodal representations.

The removal of either the auxiliary classification loss (w/o Aux) or the relation disentanglement loss (w/o Dec) also reduces overall performance. Without the auxiliary classification loss, intermediate supervision is unavailable, weakening feature learning during training. Removing the relation disentanglement loss reduces the separation among the shared, discrepant, and complementary representations, allowing redundant semantic information to interfere with each other and further degrading detection performance.

Overall, the proposed modules and loss functions complement one another and jointly improve feature representation learning and multimodal information fusion. The complete ABOOA-CDF model achieves the best results across Accuracy, Precision, Recall, and F1-score, further demonstrating the effectiveness and rationality of the proposed architecture.

### 4.4. External Dataset Validation Analysis

To further evaluate the generalization capability of the proposed ABOOA-CDF, additional experiments were conducted on the publicly available Weibo dataset. Compared with the dataset constructed in this study, Weibo contains news collected from different sources and exhibits distinct event distributions, textual styles, and cross-modal semantic characteristics. It has been widely adopted as a benchmark for multimodal fake news detection. Evaluating the proposed approach on this benchmark provides a more objective assessment of its robustness under unseen data distributions. MCAN, SpotFake+, MCNN, EANN, BCMF, and SAFE were selected as comparison methods. Accuracy, Precision, Recall, and F1-score were used as evaluation metrics. The experimental results are reported in Table 5. The bold indicates the best performance.

Overall, the baseline models show relatively similar performance. This is partly because all methods use the same multimodal input features and training settings, so their differences mainly stem from subsequent cross-modal interaction and fusion strategies rather than from the underlying feature representations. In addition, the dataset’s textual and visual information provides useful discriminative cues, enabling different multimodal models to achieve consistently strong results within a relatively narrow range. Although the Accuracy values are close, clear trade-offs remain between Precision and Recall. This indicates that the main differences among the models lie in class-specific recognition and false-positive control rather than being driven solely by class distribution.

As shown in Table 5, the competing methods exhibit relatively similar overall performance, though different trade-offs are evident across the evaluation metrics. MCAN mainly relies on cross-modal attention to capture interactions between text and images. However, its ability to model fine-grained semantic relationships remains limited in complex scenarios, leading to the lowest overall performance. SpotFake+ and MCNN improve detection by jointly exploiting textual and visual information, but they do not explicitly model different types of cross-modal relationships, which restricts further performance gains. EANN alleviates event bias through adversarial learning and achieves relatively stable results across different events. BCMF obtains the highest precision, indicating a lower false-positive rate, but its relatively low Recall suggests that more fake news samples are missed. SAFE achieves balanced performance across all evaluation metrics and is among the strongest baseline methods.

The proposed ABOOA-CDF achieves the best overall performance on the Weibo dataset. It obtains an Accuracy of 93.40 ± 0.93%, a Recall of 93.26 ± 1.29%, and an F1-score of 93.76 ± 0.91%, outperforming all competing methods on these three metrics. Its Precision reaches 94.27 ± 0.85%, which is only slightly lower than the 94.69 ± 0.86% achieved by BCMF. Compared with SAFE, the strongest overall baseline, ABOOA-CDF improves Accuracy, Recall, and F1-score by 0.53%, 0.42%, and 0.50%, respectively. These results indicate that the proposed method maintains high Precision while identifying a larger proportion of fake news, resulting in more balanced detection performance.

The superior performance of ABOOA-CDF can be attributed to the complementary contributions of its three core components. The Adaptive Binary Osprey Optimization Algorithm effectively removes redundant multimodal features while preserving discriminative information prior to feature fusion. The Cross-modal Relation Disentanglement Module explicitly separates shared, discrepant, and complementary semantic information, enabling more comprehensive modeling of interactions between text and images. Furthermore, the Adaptive Semantic Fusion Module dynamically adjusts each relation’s contribution based on the characteristics of individual samples, leading to more effective multimodal representation learning. These components work together to reduce dependence on a specific data distribution and enable the proposed model to maintain stable performance across different datasets. Therefore, the experiments on the Weibo benchmark further demonstrate the robustness and cross-dataset generalization capability of ABOOA-CDF.

### 4.5. Comparison of Feature Selection Optimization Algorithms

To evaluate the effectiveness of ABOOA for multimodal feature selection, six swarm intelligence optimization algorithms are compared: SSA, OOA, PSO, GWO, WOA, and MFO. Each algorithm is combined with the proposed cross-modal disentangled fusion framework to construct SSA-CDF, OOA-CDF, PSO-CDF, GWO-CDF, WOA-CDF, MFO-CDF, and ABOOA-CDF. For a fair comparison, all models employ the same dataset split, network architecture, and training settings. The only difference is the optimization algorithm used during feature selection. The experimental results are presented in Table 6. The bold indicates the best performance.

SSA (Sparrow Search Algorithm) performs feature selection by simulating the foraging and anti-predation behaviors of sparrows. Although it provides good global exploration, it is more likely to become trapped in local optima later in the search. PSO (Particle Swarm Optimization) updates candidate feature subsets through cooperative particle movement. It converges rapidly but may suffer from premature convergence when population diversity decreases [40]. GWO (Grey Wolf Optimizer) simulates the hierarchical hunting strategy of grey wolves. It provides effective local exploitation, although its exploration capability gradually weakens in the later optimization stage [41]. WOA (Whale Optimization Algorithm) performs optimization by mimicking the bubble-net hunting behavior of humpback whales. While it balances global exploration and local exploitation, its stability may decline in complex high-dimensional feature spaces. MFO (Moth-Flame Optimization) employs a spiral updating strategy to search for optimal feature subsets. It exhibits strong global exploration capability but generally converges more slowly than several other optimization algorithms [42]. OOA (Osprey Optimization Algorithm) performs feature selection by simulating the hunting behavior of ospreys and provides stronger local exploitation than many conventional swarm intelligence algorithms.

The proposed ABOOA extends OOA by incorporating an adaptive search mechanism and a binary optimization strategy. These improvements enhance feature selection quality and provide more informative feature representations for subsequent multimodal fusion.

Compared with the model without feature selection, all swarm intelligence optimization algorithms improve the detection performance to varying degrees. However, their search capability and feature selection quality differ considerably. SSA-CDF achieves the lowest overall performance, indicating that SSA is more likely to become trapped in local optima when searching complex feature spaces, resulting in suboptimal feature subsets. OOA-CDF and PSO-CDF outperform SSA-CDF, but their Recall and F1-score remain relatively low, suggesting that the selected features are still insufficient to represent multimodal information.

In contrast, GWO-CDF, WOA-CDF, and MFO-CDF achieve better overall performance. Among these baseline optimization algorithms, MFO-CDF produces the strongest results, achieving an Accuracy of 96.63% and an F1-score of 92.12%. This result indicates that MFO possesses strong global search capabilities and can identify high-quality feature subsets. Nevertheless, compared with ABOOA-CDF, its Accuracy, Recall, and F1-score are still lower by 1.02%, 4.17%, and 2.49%, respectively. These results suggest that conventional optimization algorithms still have limitations in refining feature subsets during the later search stage.

The proposed ABOOA-CDF achieves the best performance on all four evaluation metrics. Compared with the other optimization algorithms, ABOOA not only maintains high precision but also further improves recall, resulting in a better balance between precision and sensitivity. These improvements demonstrate that ABOOA provides more effective global exploration and local exploitation, enabling it to identify more discriminative multimodal features. The selected features provide higher-quality inputs for learning the shared, discrepant, and complementary representations, thereby further improving multimodal fake news detection performance.

### 4.6. Hyperparameter Sensitivity Analysis

**Figure 2:**
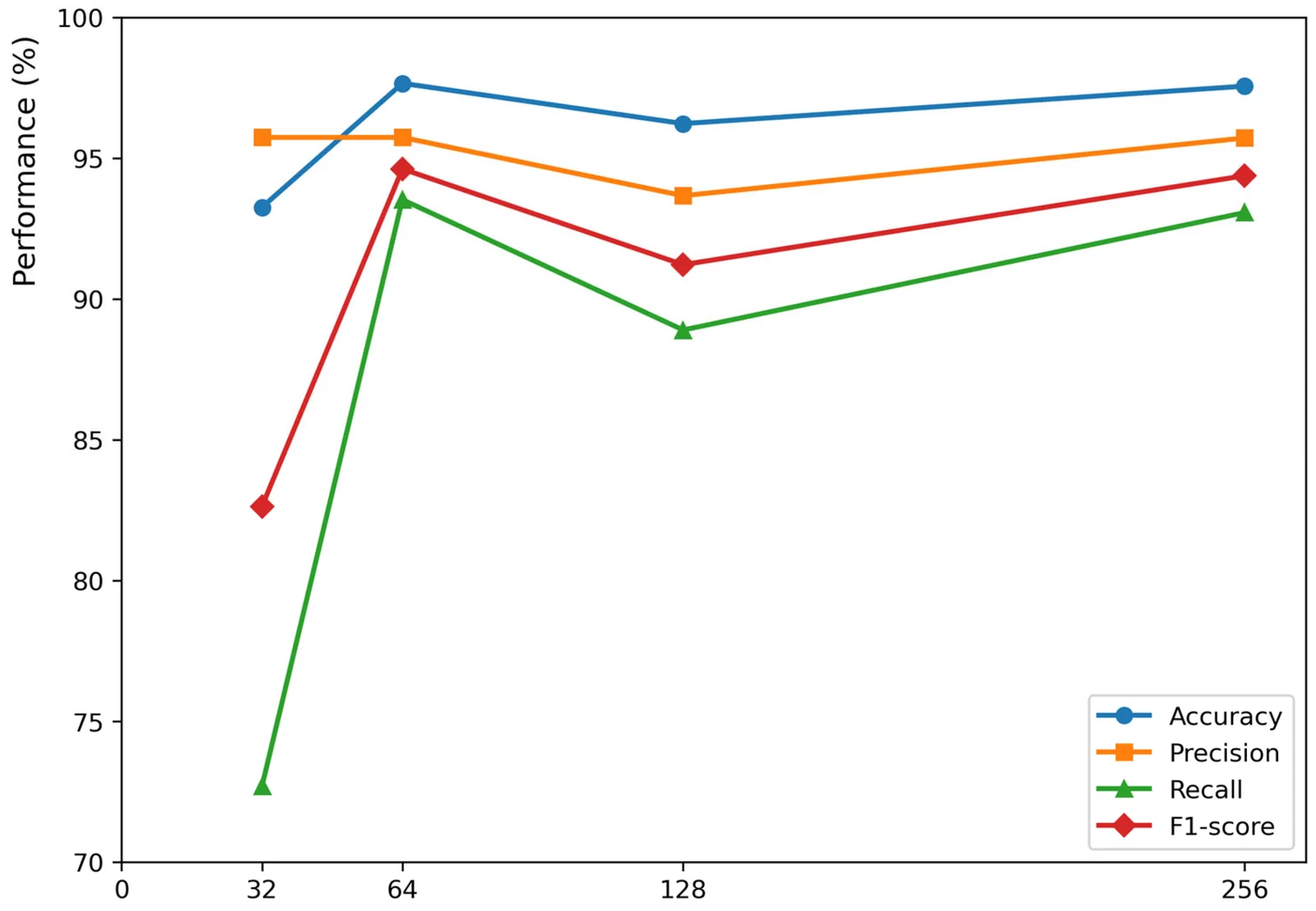
Effect of the Hidden Dimension on Model Performance.

**Figure 3:**
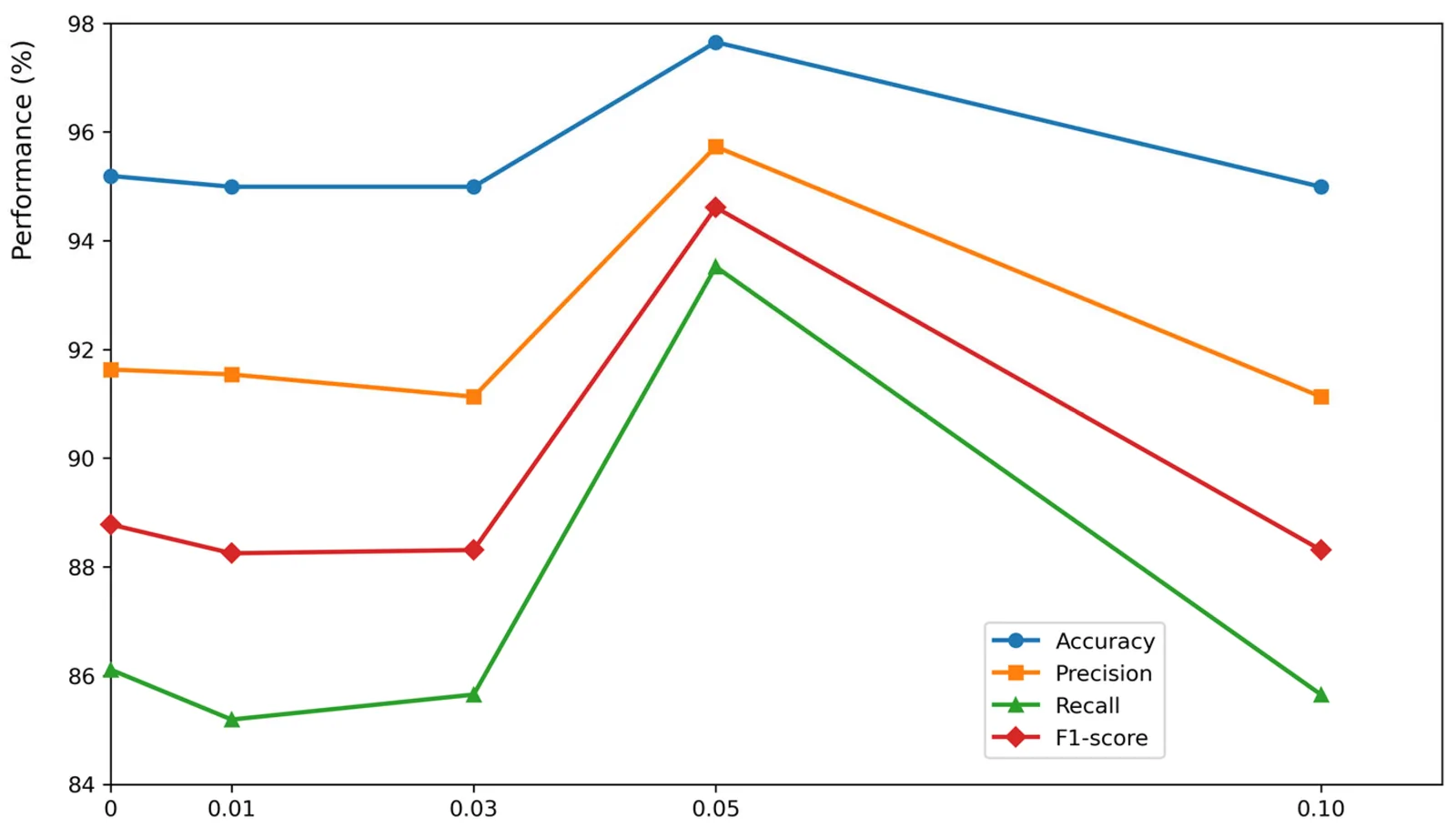
Effect of the Decoupling Loss Weight β on Model Performance.

**Figure 4:**
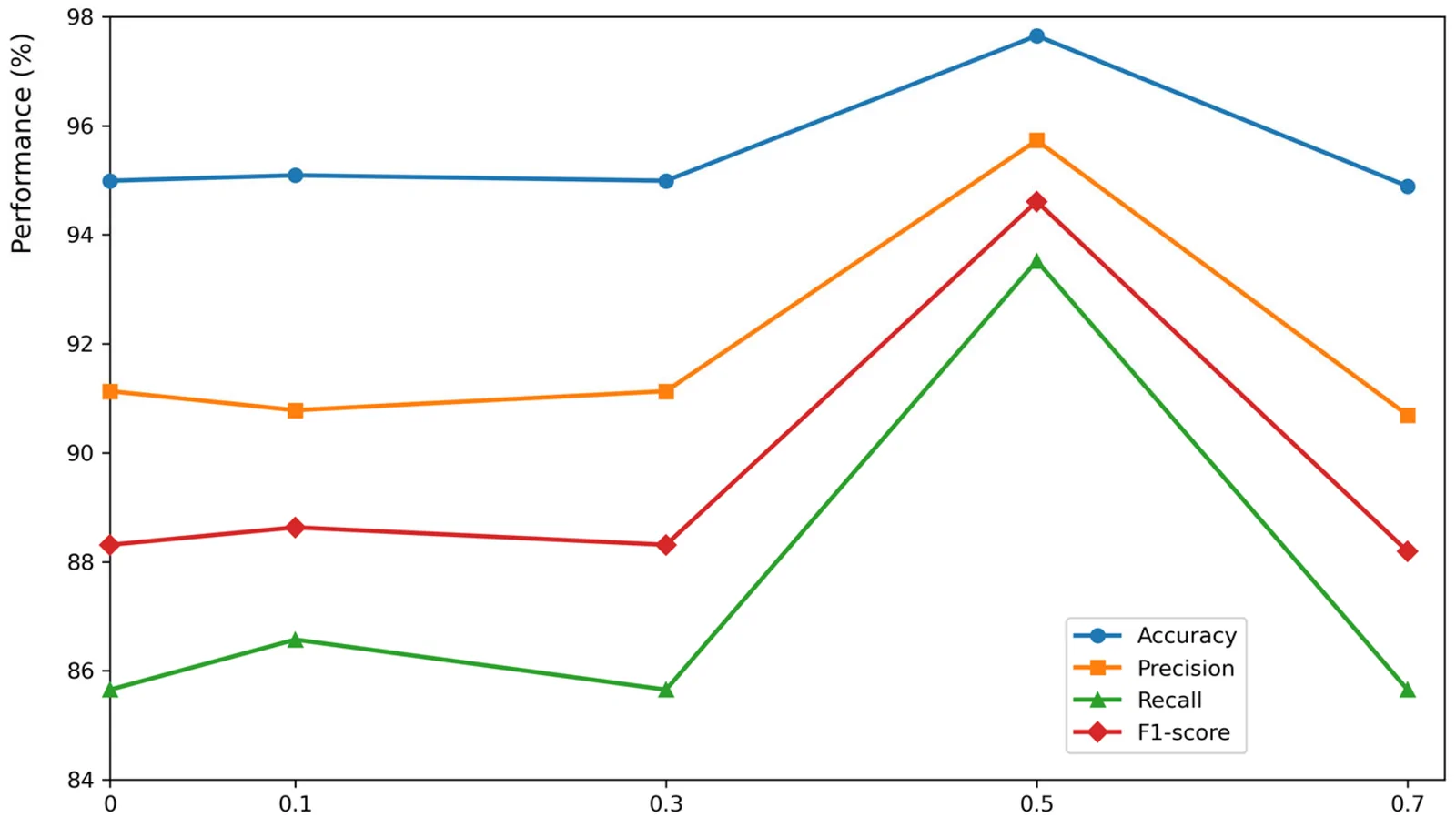
Effect of the Auxiliary Classification Loss Weight μ on Model Performance.

To investigate the sensitivity of the proposed model to key hyperparameters, experiments are conducted on the hidden dimension, decoupling loss weight β, and auxiliary classification loss weight μ. In each experiment, only one hyperparameter is varied, while all other settings remain unchanged. Accuracy, Precision, Recall, and F1-score are adopted as evaluation metrics. The corresponding results are presented in Figure 2, Figure 3 and Figure 4.

#### 4.6.1. Hidden Dimension

As shown in Figure 2, the hidden dimension has a noticeable impact on the proposed model’s performance. Increasing the hidden dimension from 32 to 64 leads to a substantial improvement across all evaluation metrics, indicating that a larger hidden representation enhances feature learning and enables more effective extraction of semantic information from both text and images. When the hidden dimension is further increased to 128, the model performance declines, suggesting that an excessively large hidden space may introduce redundant information and reduce feature learning efficiency. Increasing the hidden dimension to 256 partially recovers performance. However, the improvement remains limited. Considering both detection performance and computational cost, the hidden dimension is set to 64 in the subsequent experiments.

#### 4.6.2. Decoupling Loss Weight

As shown in Figure 3, the decoupling loss weight β significantly affects model performance. When β increases from 0 to 0.03, all evaluation metrics exhibit only minor variations and remain relatively stable. As β is further increased to 0.05, Accuracy, Precision, Recall, and F1-score all reach their highest values, indicating that an appropriate decoupling constraint improves the separation of multimodal representations and enhances feature learning. However, when β is further increased to 0.10, all evaluation metrics decline. This result suggests that an excessively strong decoupling constraint may over-restrict the learning of informative features, thereby degrading the overall classification performance.

#### 4.6.3. Auxiliary Classification Loss Weight

As shown in Figure 4, the auxiliary classification loss weight μ also influences the performance of the proposed model. When μ increases from 0 to 0.3, all evaluation metrics exhibit only slight fluctuations and remain relatively stable. As μ approaches 0.50, Accuracy, Precision, Recall, and F1-score all attain their maximum values, indicating that an appropriate auxiliary classification loss strengthens the discriminative capability of the learned representations. However, when μ is further increased to 0.70, all evaluation metrics decline. This result suggests that an excessively large auxiliary classification constraint may interfere with the optimization of the primary classification objective, thereby reducing the overall detection performance.

### 4.7. Convergence Analysis of Different Optimization Algorithms

**Figure 5:**
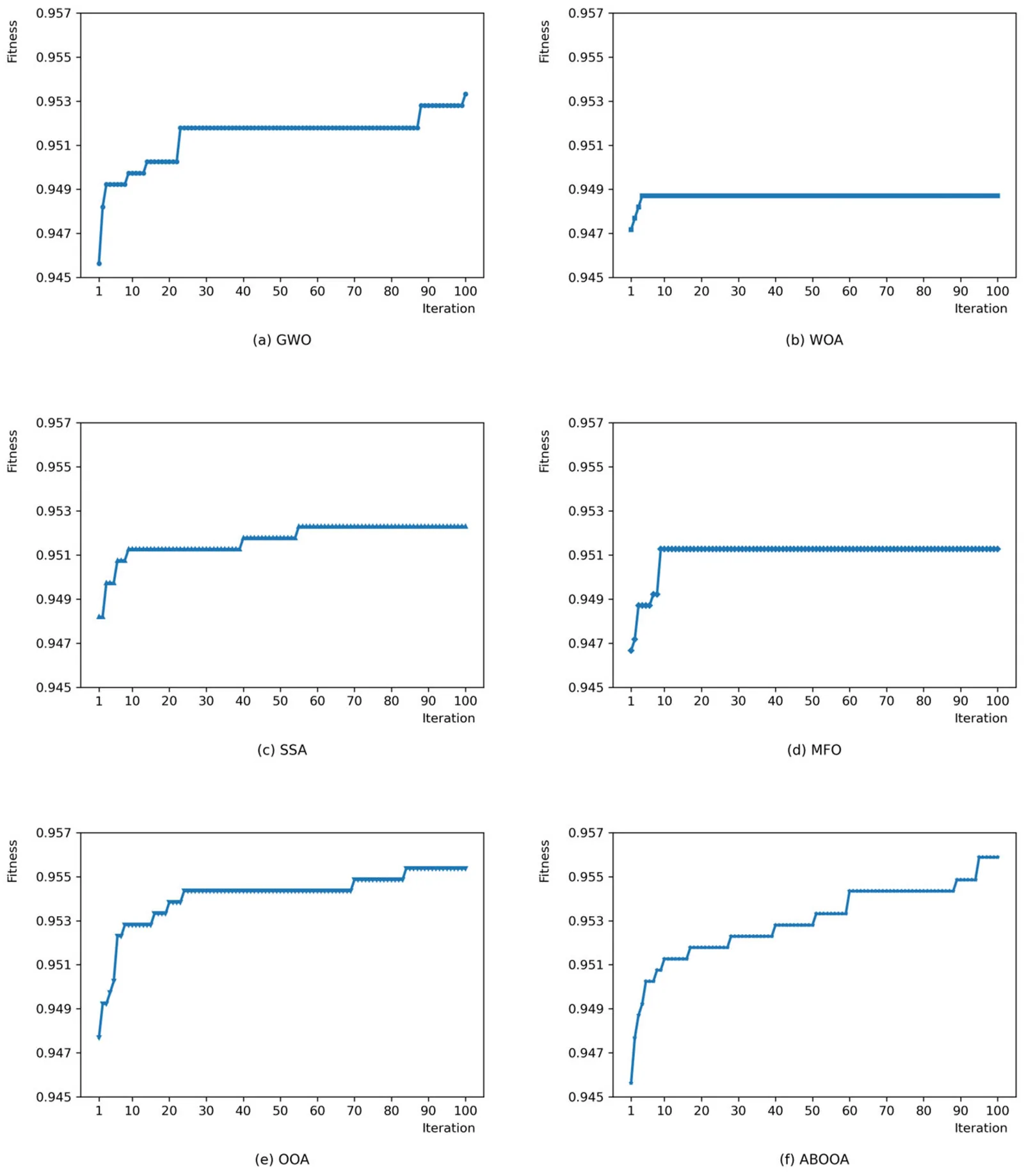
Convergence Curves of Different Optimization Algorithms.

To further evaluate the search capability of different optimization algorithms during feature selection, the convergence behaviors of GWO, WOA, SSA, MFO, OOA, and the proposed ABOOA are compared. The corresponding convergence curves are presented in Figure 5.

As shown in Figure 5, all optimization algorithms converge within a limited number of iterations, although their convergence rates and final fitness values differ noticeably. WOA, SSA, and MFO converge rapidly during the early search stage but become trapped in local optima at relatively early iterations. Consequently, their fitness values remain almost unchanged during the subsequent optimization process, indicating limited search capability. GWO and OOA continue to improve solution quality in later iterations and achieve higher final fitness values than the previous algorithms, although the overall improvement remains moderate.

In contrast, ABOOA maintains a fast convergence rate during the early iterations while continuing to search for better solutions throughout the middle and later stages. Its fitness curve exhibits several stable improvements before reaching the highest final fitness value among all compared algorithms. These results demonstrate that the proposed improvements preserve strong global exploration while significantly enhancing local exploitation. As a result, ABOOA is less likely to become trapped in local optima and can identify higher-quality feature subsets, providing more informative inputs to the subsequent multimodal fake news detection model.

### 4.8. Computational Complexity and Convergence Analysis

To assess the computational cost of ABOOA-CDF, we analyze the time complexity, space complexity, and convergence behavior of the ABOOA feature optimization stage and the CDF cross-modal representation learning stage. The CDF stage includes CRDM, ASFM, and the final classification process. The overall framework follows a sequential pipeline of feature optimization, relation modeling, and semantic fusion.

Let P denote the population size of ABOOA, T the maximum number of iterations, D the original feature dimension, and $C_{f}$ the cost of a single fitness evaluation. At each iteration, candidate prey sets are constructed according to population fitness. Comparing individuals within the population requires $O\left(P^{2}\right)$ operations. Global exploration, local exploitation, boundary handling, and adaptive binary mapping are then performed over all D dimensions of each search agent. The corresponding search-update cost is $O\left(PD\right)$. Each candidate feature subset must also be evaluated by the fitness function, resulting in $O\left(PC_{f}\right)$ additional computation. The overall time complexity of ABOOA is therefore $O\left(T\left(P^{2}+PD+PC_{f}\right)\right)$.

The main computational burden of ABOOA comes from candidate prey search and fitness evaluation. The former grows with the population size, whereas the latter depends on the cost of evaluating each selected feature subset. After feature optimization, the selected text and image features are passed to the CDF stage. Let $d_{t}$ and $d_{v}$ denote the selected text and image feature dimensions, respectively, h the dimension of the shared latent space, n the number of training samples, and E the number of training epochs. Projecting the two modalities into the common latent space requires $O\left(nh\left(d_{t}+d_{v}\right)\right)$.

CRDM then models shared, discrepant, and complementary relations. Its dominant operations are linear transformations in the latent space, yielding a computational cost of $O\left(nh^{2}\right)$. ASFM estimates adaptive fusion weights from the three relation representations and performs feature fusion and classification. Combining modality projection, relation modeling, and adaptive fusion, the time complexity of one CDF training epoch can be expressed as $O\left(nh\left(d_{t}+d_{v}+h\right)\right)$. Over E training epochs, the total complexity of CDF becomes $O\left(Enh\left(d_{t}+d_{v}+h\right)\right)$. The computational cost of CDF is mainly governed by the number of training samples, the selected feature dimensions, and the latent dimension. The input dimensions affect modality projection, while the latent dimension contributes most of the cost associated with relation modeling.

Since ABOOA and CDF are executed sequentially, the total time complexity of ABOOA-CDF is $O\left(T\left(P^{2}+PD+PC_{f}\right)+Enh\left(d_{t}+d_{v}+h\right)\right).$ The overall computation therefore consists of two distinct parts. ABOOA performs high-dimensional feature search, whereas CDF carries out cross-modal relation modeling, semantic fusion, and classification. Their respective costs are determined by the search process and the subsequent network training.

For space complexity, ABOOA maintains both the continuous position matrix and the corresponding binary feature-selection matrix. Its dominant memory requirement is $O\left(PD\right)$. The parameters in CDF mainly arise from modality projection and linear transformations in the latent space. Their storage complexity is $O\left(h\left(d_{t}+d_{v}\right)+h^{2}\right)$. Let B be the batch size. The intermediate hidden representations require approximately $O\left(Bh\right)$ additional memory during training. Hence, the overall space complexity of ABOOA-CDF is $O\left(PD+h\left(d_{t}+d_{v}\right)+h^{2}+Bh\right).$ The memory demand of ABOOA is mainly affected by the population size and feature dimension, whereas CDF is dominated by model parameters and intermediate representations. For convergence, ABOOA adopts greedy selection and replaces the current solution only when a candidate achieves a better fitness value. Thus, the best fitness satisfies $F_{best}^{t+1}\leq F_{best}^{t}$.

As the iterations proceed, the adaptive search factor gradually reduces the update amplitude, allowing the optimization process to shift from global exploration toward local exploitation and improving the stability of the later search stage. Overall, ABOOA performs feature optimization through adaptive search and greedy selection, while CDF focuses on cross-modal representation learning and semantic fusion. Together, the two stages form the optimization process of ABOOA-CDF.

## 5. Conclusions and Future Work

### 5.1. Conclusions

This paper presents a multimodal fake news detection model based on the Adaptive Binary Osprey Optimization Algorithm and Cross-modal Disentangled Fusion (ABOOA-CDF) to address feature representation and semantic fusion in this task. The proposed framework establishes a comprehensive detection pipeline comprising feature selection, cross-modal relation modeling, and adaptive semantic fusion. By integrating swarm intelligence optimization with multimodal semantic learning, the model effectively transforms raw multimodal features into reliable classification results, providing a new solution for multimodal fake news detection.

During the feature selection stage, an Adaptive Binary Osprey Optimization Algorithm (ABOOA) is developed to optimize textual and visual representations. Compared with the original Osprey Optimization Algorithm, ABOOA incorporates circle chaotic initialization, an adaptive search strategy, and an adaptive binary mapping mechanism. These improvements enable the search process to dynamically adjust its optimization behavior across different evolutionary stages. Specifically, chaotic initialization enhances the diversity of the initial population, the adaptive search strategy improves both global exploration and local exploitation, and the binary mapping mechanism effectively bridges continuous optimization and discrete feature subset selection. As a result, ABOOA generates compact and highly discriminative multimodal features for subsequent representation learning.

To model cross-modal semantic relationships, a Cross-modal Relation Disentanglement Module (CRDM) is introduced to explore interactions between images and text from multiple semantic perspectives. CRDM explicitly decomposes cross-modal representations into shared, discrepant, and complementary information, enabling the model to distinguish semantic consistency, modality-specific characteristics, and interaction-induced complementary semantics. Furthermore, the relation disentanglement constraint and auxiliary supervision encourage these representations to remain semantically distinct while preserving their discriminative capability for fake news detection.

For multimodal fusion, an Adaptive Semantic Fusion Module (ASFM) is proposed to dynamically integrate the three disentangled semantic representations generated by CRDM. Instead of applying fixed fusion coefficients, ASFM adaptively determines the contributions of shared, discrepant, and complementary information based on the semantic characteristics of each sample. This adaptive fusion strategy enables the model to exploit diverse types of cross-modal evidence better and handle complex fake news scenarios.

Experimental results demonstrate that ABOOA-CDF performs well in multimodal fake news detection, achieving superior results in Accuracy, Precision, Recall, and F1-score. In addition, the ablation study and the comparison of feature selection optimization algorithms verify the effectiveness of ABOOA, CRDM, and ASFM. These findings indicate that feature optimization, relation disentanglement, and adaptive semantic fusion complement each other and jointly improve the overall detection capability. The proposed framework provides an effective and systematic approach to multimodal fake news detection and serves as a useful reference for information authenticity analysis in complex online environments.

### 5.2. Limitations and Future Work

Although ABOOA-CDF demonstrates promising performance in feature selection, cross-modal relation modeling, and semantic fusion, there is still room for improvement in more challenging application scenarios and practical deployment. Future research should focus on the following directions.

First, the generalization capability of the proposed model should be further investigated in more complex multi-source multimodal environments. Evaluation mainly relies on standard dataset splits and therefore reflects performance under relatively consistent training and testing distributions. In real-world social media, however, news content changes over time, while event semantics, propagation patterns, and cross-modal relationships may also shift. Future work will consider datasets with explicit temporal information and adopt stricter Out-of-Distribution (OOD) evaluation protocols, such as temporal splitting. This will allow a more systematic assessment of ABOOA-CDF under temporal distribution shifts and provide further evidence regarding the robustness of cross-modal relation disentanglement in CRDM. Beyond distribution shifts, the current framework mainly focuses on textual and visual information. Additional modalities, including audio, video, and user propagation behavior, could be incorporated to investigate collaborative representation learning across heterogeneous sources. Cross-lingual semantic representation is another promising direction for examining model adaptability across different languages and cultural contexts, further extending its generalization to more diverse information environments.

Second, recent advances in large language models (LLMs) and vision-language models (VLMs) offer new opportunities to improve multimodal fake news detection. Future research should investigate how to incorporate their rich semantic knowledge and reasoning capability into ABOOA-CDF to strengthen semantic understanding and cross-modal relation modeling. The prior knowledge provided by pretrained models can help the framework better understand event backgrounds, entity relationships, and implicit semantic information. In addition, integrating knowledge-enhanced learning strategies may further improve the interpretability of the detection results, enabling the model not only to determine the authenticity of news but also to provide more intuitive and reliable explanations for its predictions.

Overall, ABOOA-CDF has demonstrated strong effectiveness and robustness in feature optimization, cross-modal relation disentanglement, and adaptive semantic fusion. Future work should continue to improve the model across generalization, knowledge-enhanced learning, and multimodal reasoning, advancing multimodal fake news detection toward more intelligent, reliable, and scalable solutions.

## Figures and Tables

**Table 1:** Data Sources and Sample Distribution.

Sources	Text Samples	Images
Sina News	2449	1260
Science China	4186	4100
China Internet Joint Rumor Refutation Platform	903	902

**Table 2:** Main Hyperparameter Settings.

Hyperparameters	Settings
Batch Size	32
Epochs	30
Learning Rate	5 × 10^−6^
Hidden Dimension	64
Auxiliary Loss Weight μ	0.50
Decoupling Loss Weight β	0.05

**Table 3:** Comparison Results of Multimodal Fake News Detection Models.

Models	Accuracy (%)	Precision (%)	Recall (%)	F1-Score (%)
MCAN	95.32 ± 0.45	91.94 ± 1.54	86.39 ± 0.84	89.07 ± 1.00
MCNN	95.46 ± 0.53	91.52 ± 1.91	87.59 ± 0.89	89.50 ± 1.16
BCMF	95.48 ± 0.51	88.90 ± 1.86	90.93 ± 1.37	89.89 ± 1.10
EANN	95.56 ± 0.28	91.69 ± 0.53	87.87 ± 0.89	89.74 ± 0.66
SpotFake+	95.73 ± 0.28	91.84 ± 0.74	88.52 ± 0.60	90.15 ± 0.65
SAFE	95.91 ± 0.26	89.96 ± 1.40	91.76 ± 1.44	90.83 ± 0.57
**ABOOA-CDF**	**96.93 ± 0.56**	**93.00 ± 1.97**	**93.15 ± 0.95**	**93.07 ± 1.23**

**Table 4:** Results of the Component Ablation Experiment.

Models	Accuracy (%)	Precision (%)	Recall (%)	F1-Score (%)
w/o C	95.50	92.16	87.04	89.52
w/o D	96.22	94.09	88.43	91.17
w/o M	95.40	89.40	89.81	89.61
w/o ASFM	95.19	88.24	90.28	89.24
w/o Aux	95.19	86.90	92.13	89.44
w/o Dec	94.99	85.53	93.06	89.14
**ABOOA-CDF**	**97.65**	**95.73**	**93.52**	**94.61**

**Table 5:** Cross-Dataset Generalization Performance on the Weibo Dataset.

Models	Accuracy (%)	Precision (%)	Recall (%)	F1-Score (%)
MCAN	91.95 ± 0.89	92.44 ± 1.96	92.48 ± 1.97	92.43 ± 0.83
SpotFake+	92.45 ± 0.52	93.58 ± 1.67	92.19 ± 2.32	92.85 ± 0.57
MCNN	92.54 ± 0.71	93.67 ± 1.68	92.26 ± 2.02	92.93 ± 0.70
EANN	92.72 ± 0.70	93.97 ± 0.70	92.26 ± 2.09	93.09 ± 0.76
BCMF	92.85 ± 0.66	**94.69 ± 0.86**	91.72 ± 1.79	93.17 ± 0.69
SAFE	92.87 ± 0.72	93.69 ± 0.66	92.84 ± 1.14	93.26 ± 0.70
**ABOOA-CDF**	**93.40 ± 0.93**	94.27 ± 0.85	**93.26 ± 1.29**	**93.76 ± 0.91**

**Table 6:** Performance Comparison of Different Feature Selection Optimization Algorithms.

Models	Accuracy (%)	Precision (%)	Recall (%)	F1-Score (%)
SSA-CDF	94.99	91.13	85.65	88.31
OOA-CDF	94.99	89.57	87.50	88.52
PSO-CDF	95.30	92.50	85.65	88.94
GWO-CDF	96.22	93.66	88.89	91.21
WOA-CDF	96.32	95.00	87.96	91.35
MFO-CDF	96.63	95.07	89.35	92.12
**ABOOA-CDF**	**97.65**	**95.73**	**93.52**	**94.61**

## Data Availability

The original code and data presented in the study are openly available in GitHub at https://github.com/hao0809-oss/ABOOA-CDF (accessed on 21 July 2026).
